# The emerging role of exosomal miRNAs as a diagnostic and therapeutic biomarker in *Mycobacterium tuberculosis* infection

**DOI:** 10.1186/s10020-021-00296-1

**Published:** 2021-04-01

**Authors:** Rasoul Mirzaei, Sajad Babakhani, Parisa Ajorloo, Razieh Heidari Ahmadi, Seyed Reza Hosseini-Fard, Hossein Keyvani, Yaghoub Ahmadyousefi, Ali Teimoori, Farhad Zamani, Sajad Karampoor, Rasoul Yousefimashouf

**Affiliations:** 1 Department of Microbiology, School of Medicine, Hamadan University of Medical Sciences, Hamadan, Iran; 2 Venom and Biotherapeutics Molecules Lab, Medical Biotechnology Department, Biotechnology Research Center, Pasteur Institute of Iran, Tehran, Iran; 3 Department of Microbiology, North Tehran Branch, Islamic Azad University, Tehran, Iran; 4 Department of Biology, Sciences and Research Branch, Islamic Azad University, Tehran, Iran; 5 Department of Genetics, Faculty of Advanced Sciences and Technology, Tehran Medical Sciences Islamic Azad University, Tehran, Iran; 6 Department of Biochemistry, School of Medicine, Tehran University of Medical Sciences, Tehran, Iran; 7 Gastrointestinal and Liver Diseases Research Center, Iran University of Medical Sciences, Tehran, Iran; 8 Department of Virology, School of Medicine, Iran University of Medical Sciences, Tehran, Iran; 9 Department of Medical Biotechnology, School of Advanced Medical Sciences and Technologies, Hamadan University of Medical Sciences, Hamadan, Iran; 10 Research Center for Molecular Medicine, Hamadan University of Medical Sciences, Hamadan, Iran; 11 Department of Virology, School of Medicine, Hamadan University of Medical Sciences, Hamadan, Iran

**Keywords:** Tuberculosis, Exosomes, Exosomal miRNA, Diagnostic, Therapeutic biomarker

## Abstract

Tuberculosis (TB), caused by *Mycobacterium tuberculosis* (Mtb), has been the world’s driving fatal bacterial contagious disease globally. It continues a public health emergency, and around one-third of the global community has been affected by latent TB infection (LTBI). This is mostly due to the difficulty in diagnosing and treating patients with TB and LTBI. Exosomes are nanovesicles (40–100 nm) released from different cell types, containing proteins, lipids, mRNA, and miRNA, and they allow the transfer of one’s cargo to other cells. The functional and diagnostic potential of exosomal miRNAs has been demonstrated in bacterial infections, including TB. Besides, it has been recognized that cells infected by intracellular pathogens such as Mtb can be secreting an exosome, which is implicated in the infection’s fate. Exosomes, therefore, open a unique viewpoint on the investigative process of TB pathogenicity. This study explores the possible function of exosomal miRNAs as a diagnostic biomarker. Moreover, we include the latest data on the pathogenic and therapeutic role of exosomal miRNAs in TB.

## Introduction

*Mycobacterium tuberculosis* (Mtb), a TB causative agent, is one of the world's major deadly contagious illnesses (Dye and Williams 2010). Current figures suggest that nearly one-fourth of all people worldwide have been afflicted with Mtb and that TB causes 1.4 million deaths per year (Organization WH 2019). Also, approximately 2 billion people are latently infected with Mtb. Only 5–10% of infected people can produce active TB in their lifespan, which happens whenever the immune reaction can no further hold the bacterium (Tufariello et al. 2003). The novel biomarkers' development is necessary for the early diagnosis of TB (for monitoring and mitigating infection transmission) since the current diagnostic approaches for TB encounter difficulties (Velayati et al. 2015, 2016) Exosomes have been suggested as experimental medical biomarkers for various pathological disorders, such as tumors and infectious diseases (Velayati et al. 2015; Sadri Nahand et al. 2020; Nahand et al. 2019). Exosomes are 30–150 nm in size and basically produced from most human cells into the lymphatic organ and blood to promote cell-to-cell contact by moving separate fragments from donor to receiver cells (Alipoor et al. 2016a). These host vesicles containing lipids, nucleic acids, and proteins originated from cells, indicating cell defects and providing useful knowledge on the disorder, including TB (Schorey and Bhatnagar 2008).

MicroRNAs, also called miRNAs, are small 18–22 nt RNAs that significantly modify gene expression and transcription (Mirzaei et al. 2020, 2021). miRNAs can influence most physiological purposes, and their disturbances are correlated with a different pathological condition (Alipoor et al. 2016b). Functional miRNAs can be surrounded within the exosomes, transferred to target cells, altering the receiver cell role by modifying their transcriptome and proteome (Alipoor et al. 2016a). miRNAs are implicated in the direction of inflammatory means throughout Mtb infection (Alipoor et al. 2017; Furci et al. 2013). Mtb infection causes a sequence of biochemical responses in infected cells, driving host cell metabolic reprogramming and thereby immune dysregulation of host cells (Moschos et al. 2007). These host cell roles modifications facilitate bacteria to expropriate vital host determinants to provide their requirements to permit intracellular endurance (Mehrotra et al. 2014). These methods may be regulated by the demolition of host miRNA arrangements implicated in managing metabolism (carbon, lipid, and nitrogen) in the infected host cells (Eisenreich et al. 2013; Smith 2003). In this work, we will summarize and describe the knowledge available on the human immune response to TB, the dynamics of the host–pathogen interaction, and illustrate the significance of the signal transduction pathways implicated in TB pathophysiology. Besides, we evaluated the possible function of exosomal miRNA as a diagnostic biomarker. Moreover, we also include the latest data on the pathogenic and therapeutic function of exosomal miRNA in TB.

## Immunopathogenesis of *Mycobacterium tuberculosis* infection

**Fig. 1:**
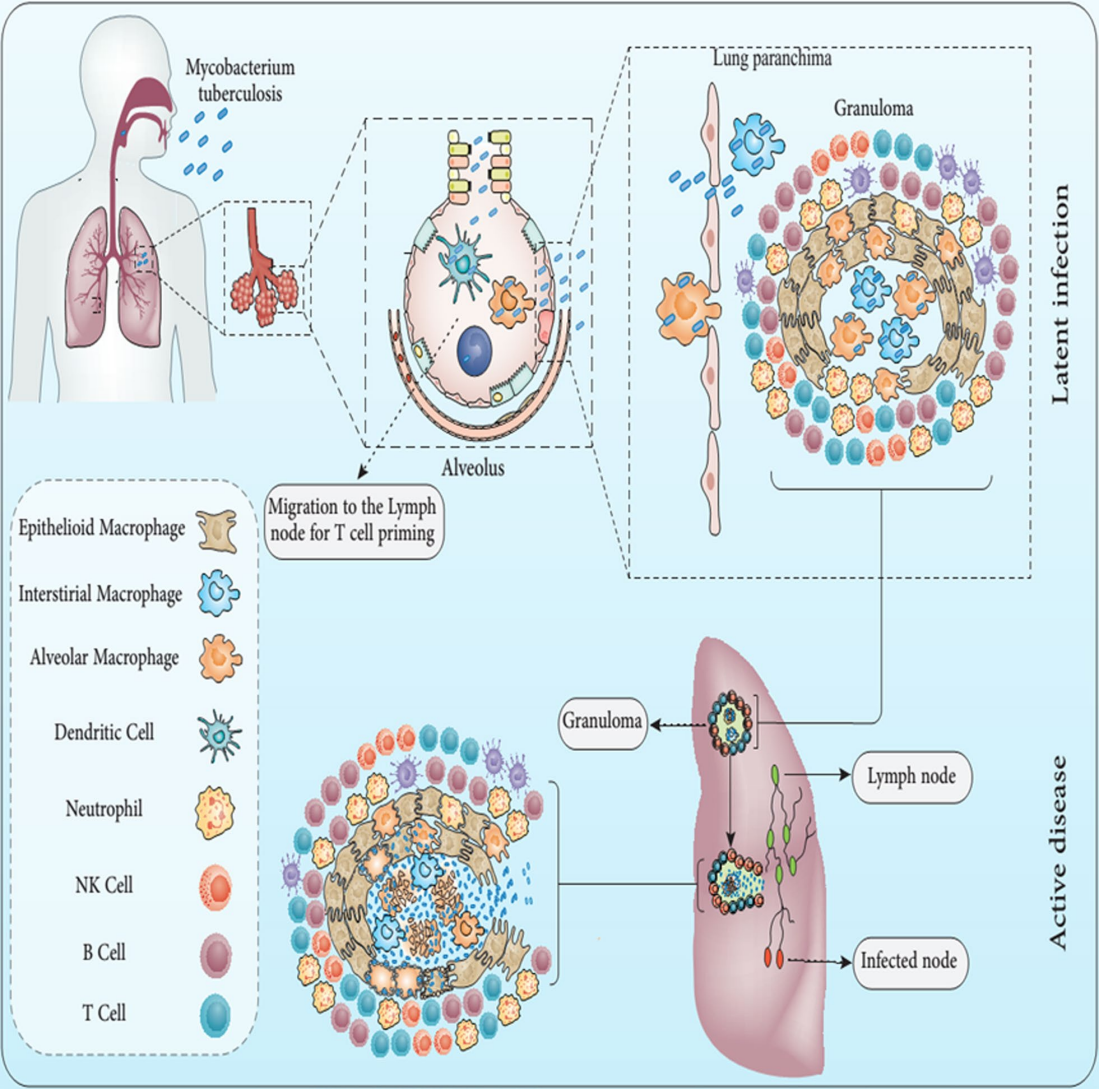
Immunopathogenesis of tuberculosis. Infection occurs when Mtb enters the lung through the respiratory tract and then arrives in the alveolar space, where it faces macrophages residing in the alveolar space. Suppose this part of the immune system fails to kill Mtb. In that case, this bacteria attacks the interstitial tissue of the lung, which either infects the lung epithelial cells directly or is transmitted to the lung parenchymal tissue through the infected macrophages. Afterward, DCs or inflammatory monocytes lead to the transfer of Mtb to the lymph nodes of the lung for priming of T cells. The alliance of these events triggers the recruitment of immune cells, including B and T cells, to the lung parenchyma, resulting in granuloma formation. When the bacterial mass becomes too high, the granuloma can no longer control the infection, so the bacteria spread to other tissues, including the brain. At this stage, the bacteria can enter the bloodstream or re-enter the respiratory tract to be finally released. At this time, the infected host can transmit the infection to others, which is called active tuberculosis (Pai et al. 2016). Mtb: *Mycobacterium tuberculosis*; DCs: dendritic cells

The immune system reactions to TB are a vigorous response to robust pathogen attack (Fig. 1) (Mortaz et al. 2012). This relationship with the cellular immune response occurs in a complex setting involving a broad spectrum of proinflammatory cytokines. These different influences significantly affect the body's capacity to suppress infection virtually (Mortaz et al. 2012). In the current years, various experiments have started to supplement human studies utilizing bronchoalveolar lavage (BAL) content from TB patients and healthy controls volunteers (Mortaz et al. 2012).

In this respect, the evolving evidence points out that the host defends towards Mtb requires cellular response usually mediated by T helper (Th) type 1 (Th1)/Th 17 cells (Th17) (Mortellaro et al. 2009; Schluger and Rom 1998). It has been demonstrated for an extended period that the T-lymphocyte subgroup centered on immune reactions has been identified as the Th1/Th 2, which offers a basis for knowing how the human immune system responds to various pathogens (Mortaz et al. 2012; Mortellaro et al. 2009; Schluger and Rom 1998; Rasoul et al. 2019). The two main subgroups of CD4 Th lymphocytes, Th1 and Th2, have separate mediator development profiles and perform different functions in immune function (Mortaz et al. 2012; Mortellaro et al. 2009; Schluger and Rom 1998; Mirzaei et al. 2017). Th1 is defined by interleukin-2 (IL-2), tumor necrosis factor-alpha (TNF-α), interferon-gamma (IFN-γ) development, and Th2 cells by Interleukin-4 (IL-4), interleukin-13 (IL-13), and interleukin-5 (IL-5) formation (Mortellaro et al. 2009; Schluger and Rom 1998). Th1 cytokines promote macrophages (MQs) and cell-mediated responses that are critical for immunity to intracellular pathogens as well as delayed-type hypersensitivity (DTH) and cytotoxic activities (Mortellaro et al. 2009; Schluger and Rom 1998). Th2 cells produce Interleukin-6 (IL-6), IL-4, IL-5, Interleukin-10 (IL-10), and IL-13 that stimulate varied antibody development and thus frequently known to be correlated with antibody responses, which are critical in the battle against extracellular pathogens (Coffman and Romagnani 2013). Th1 and Th2 cells are strictly suppressive; IL-10, the outcome of Th2 cells, suppresses the Th1 development by working on APC (antigen-presenting cells), while the expansion of Th2 cells was blocked by IFN-γ, which produced by Th1 cells (Mosmann and Sad 1996; Romagnani 1991). In the sequence mentioned above, it has also been designated that deficiencies in the IFN-γ Th1 effector's formation have resulted in a vulnerability to TB infection (Sieling and Modlin 1994; Suzuki et al. 1989). Mtb instantly can infect inflammatory cells, notably MQs and dendritic cells (DCs) (Kornfeld et al. 1999). Lipid-rich external molecules enclose this pathogen as an envelope that preserves it from toxic radicals and hydrolases generated as protection against inflammatory cells as well as MQs (Niederweis et al. 2010). Mtb can enter surrounding resting MQs as well as other cells required for proliferation (Niederweis et al. 2010). MQs, triggered by contact with aerosolized objects, have a potent microbicidal action that could destroy bacilli and avoid TB disease, but they are typically incapable of eradicating it (Ahmad 2011). Other crucial antimicrobial immunity processes involve the devastation of infected cells through cytotoxic T cells (Brighenti and Andersson 2010).

It has been found IFN-γ is the crucial cytokine for a protective immunity toward Mtb (Ahmad 2011). In this regard, defective IFN-γ and/or IFN-γ receptor (IFN-γ-R) cases further susceptible to Mtb infection (Chan and Flynn 2004; Cooper 2009; Flynn et al. 1993). The IFN-γ, generated by CD8+, CD4+ T lymphocytes, as well as NKCs along with TNF-α and, stimulates MQs to degrade the intracellular bacteria (Ahmad 2011). A critical effector process involved in the antibacterial properties of IFN-γ along TNF-α is triggering of nitric oxide (NO) and other reactive nitrogen intermediates formation by MQs through Inducible nitric oxide synthase (iNOS) (Chan and Flynn 2004; Cooper 2009; Flynn et al. 1993). Nevertheless, several Mtb components like the 19-kDa lipoprotein could attenuate the MQs stimulation toward IFN-γ via blocking of the IFN-γ-responsive genes (Pai et al. 2003, 2004; Gehring et al. 2003).

MQs, DCs, and T lymphocytes generate TNF-α, another cytokine with a crucial protective immune activity toward Mtb infection (Bean et al. 1999; Keane 2005). Besides, conversely, TNF-α involves in the immunopathology mediated TB (Flynn and Chan 2005). In vivo, mice deficient in TNF-α and/or TNF-α receptors (TNF-α-R) are further susceptible to infection (Bean et al. 1999; Keane 2005). This cytokine also induces cell migration and microbicidal granulomas production, while disruption of TNF-α activity cause overgrowth of the Mtb (Chan and Flynn 2004; Cooper 2009; Flynn et al. 1993). TNF-α generated by MQs and T lymphocytes is crucial for sufficient and prolonged protection toward Mtb infection (Saunders et al. 2005). It has been found that the phenolic glycolipid from a hyper-virulent strain of Mtb suppresses the proinflammatory cytokines IL-6, TNF-α, as well IL-12 formation by MQs (Reed et al. 2004). The role of IL-12 is also documented from enhanced susceptibility of mice and humans because individuals with defects in the IL-12 formation are much susceptible to active TB (Lichtenauer-Kaligis et al. 2003).

Complete elimination of Mtb needs a balance between inflammatory reactions (Davis and Ramakrishnan 2009). MQs are reached to the site via secreted mycobacterial components like Early secretory antigenic target-6 (ESAT-6), and infected MQ could enter and leave the producing granuloma, cause bacterial proliferation, and disseminate the infection (Tang and Sails 2015; Volkman et al. 2010). Control of TNF formation is crucial to allow MQs to be stimulated to degrade intracellular Mtb. Insufficient TNF allows enhanced proliferation inside MQs. On the other hand, further, TNF results in necrosis of MQ. Subsequently, the produced necrotic debris components generate the caseum in the center of the granuloma, allowing the exuberant extracellular proliferation of mycobacterial cells (Ramakrishnan 2012). Mycobacterial antigens via uptake into the MQ cells are delivered to the antigen-processing system called Class II major histocompatibility complex molecules (MHC class II) (Ramakrishnan 2012; Hopewell 1994). Then, Mycobacterial antigens are delivered to and stimulated Mtb-specific CD4 lymphocytes of Th1 that protect against infection by stimulating MQ via IFN-γ and killing infected immune cells (Ramakrishnan 2012; Hopewell 1994). In this regard, lysed infected MQ cells liberate mycobacteria to be destroyed via more proficient monocyte cells. Additionally, T lymphocytes directly kill mycobacterial cells via perforin and granulysin (Stenger et al. 1999).

Although CD4^+^ T cells are widely recognized as critical, other T-cell subsets, such as γδ T cells, Th17 cells, and traditional CD8^+^ T lymphocytes, have been identified through several evaluations (Barker et al. 2009; North and Jung 2004; Kaufmann 2002). In this regard, the primary growth of Mtb causes a DTH reaction defined by small necrotic sores production with dense caseous cores in host infected areas (Hanif et al. 2010). Mtb proliferation is seen to be limited when encompassed in these granulomas lesions. Granulomas comprise T cells and mononuclear phagocytes of varying maturation and stimulus stages (Flynn et al. 2011). After driving DTH and tubercle production development, activation of the MQ cells by CD4^+^ T cells allows destroying the bacilli within the tubercle lesions (Flynn et al. 2011). MQ stimulation tends to be the core stage of the acquired resistance to Mtb infection (Gideon and Flynn 2011). MQ stimulation is accomplished by T cells, which seem to be the main conciliator of the Cell-mediated immunity (CMI) response to Mtb infection (Gideon and Flynn 2011). CD4^+^  T lymphocytes mainly assist T cells that produce various forms of mediators implicated in MQ stimulation (Kaufmann 1991). In the DTH reaction to Mtb infection, CD4^+^ T lymphocytes prevail beyond CD8^+^ T lymphocytes, and they are cytotoxic cells that destroy the target cells (Kaufmann 1991). These cells are essential to hire and trigger additional monocytes/MQs for tubercle sores (Kaufmann 1991). As shown above, Th1 cells generate IFN-γ and IL-2, which seem to be trigger antimycobacterial response and are critical for the DTH reaction (Kaufmann 1991). IFN-γ directly induces MQs and enables them to eat and destroy Mtb more efficiently (Philips and Ernst 2012). MQs stimulation by cytokines gives only a limited clarification of tolerance to Mtb infection (Philips and Ernst 2012). A reaction mediated to Major Histocompatibility Complex (MHC) class I to mycobacterial disease was identified in many T cells (Philips and Ernst 2012). CD8^+^ T lymphocytes result in the activation of the MQs by developing IFN-γ (Philips and Ernst 2012). CD8^+^ T lymphocytes can also have a cytotoxic role that allows them to detect the Mtb antigens provided via MHC Class I on the surfaces of infected MQs (Philips and Ernst 2012). CD8^+^ CTLs are crucial for the killing of intracellular Mtb existing in infected MQs (Lin and Flynn 2015). CTLs affinity for Mtb antigens (Ags) has also been established in the mouse models of TB (Lin and Flynn 2015). In this line, it has been shown that exogenous antigens can apply directly to the preparation and representation pathways of Class I and produce a CD8^+^ T-cell reaction in vivo (Lin and Flynn 2015). Additionally, it has been shown that Mtb can live inside MQs, provide metabolic antigens for manufacturing, and display MHC-I on the MQ's surface (Philips and Ernst 2012). It has been proposed that Mtb could prevent processing inside the phagolysosomal circumstances (Turner and Dockrell 1996). By that means, the antigen may insert the endogenous antigen-processing route and be conferred to CD8^+^ T lymphocytes (Turner and Dockrell 1996). Consequently, it has now been hypothesized that CD4^+^ T lymphocytes also can play a key role in protective immunity against Mtb infection by cytolytic operation (Canaday et al. 2001). In this respect, CD4^+^ T lymphocytes can be lysing infected MQs, results in Mtb growth inhibition (Canaday et al. 2001). CD4^+^ T lymphocytes were observed only in vitro by human peripheral blood mononuclear cells (Mutis et al. 1993). Gamma/delta T lymphocytes have been shown to play a significant role in protecting the host against Mtb (Mutis et al. 1993). Of note, these cells could lyse MQs carrying Mtb and are a primary source of IFN-γ (Dieli et al. 2000). They may have a part to play in the early, innate immunity against Mtb since their number is magnified by Mtb and their composites in tissues (Dieli et al. 2000). Intriguingly, it has been shown that a crucial proportion of the mycobacteria-reactive gamma/delta T cell community has been induced following subcutaneous and aerosol immunization of mice with mycobacteria (Ladel et al. 1995). These cells can also release IFN-γ, which can be a significant stimulus in the initial step of the TB immune response (Ladel et al. 1995). Natural killer (NK) cells also may play a vital role in the host's immunity to Mtb, and these cells are capable of lysing Mtb -infected host cells. TB pathogens were implying functional similarities to unique CTLs (Culley 2009; Bozzano et al. 2014). The function of T cell regulator (Tregs) in immunity to Mtb has still not been identified, but, regarding human research, it has been seen that the amount of Tregs is growing in the blood and at sites of infection in active TB patients (Chen et al. 2007; Guyot-Revol et al. 2006). Ultimately, the function of Th17 cells and interleukin-23 (IL-23) has emerged recently. In this regard, it has been found that, through TB aerosol infection, the deficiency of IL-23 drives to ablation of the Th17 reactions and substantial lack of interleukin 17 (IL-17) expression in the lung (Khader et al. 2005). These data designate that IL-23 is crucial for both the Th17 community and the IL-17 reaction to TB infection (Khader et al. 2005). Regarding the function of IL-23, this cytokine is necessary for the induction of Th17 (Ag-specific) responses to Mtb infection in humans (Khader et al. 2005). IL-17 has shown to be an inflammatory cytokine causing chemokine slopes and initiating inflammation, mainly in the human lung (Sergejeva et al. 2005; Kolls and Lindén 2004; Miyamoto et al. 2003). IL-23 and IL-17 caused neutrophils and hemostasis in the mice lung infected with Mtb (Stark et al. 2005). It is also evident that IL-23 and IL-17 are working dynamically to regulate TB-induced inflammation (Stark et al. 2005). This is not remarkable because IL-17 will act as a negotiator for the collection of MQs and the initiation of (C-X-C motif) ligand (CXCL) chemokines holding IL-17-correlated promoter elements (Sergejeva et al. 2005; Khader et al. 2007; Shen et al. 2006). These data intimate that IL-17 can interfere with the aggregate of both polymorphic and mononuclear cells throughout TB infection.

## Mycobacterium derived extracellular vesicles

All other living species, including prokaryotes and eukaryotes, employ various techniques to secrete their essential molecules into the extracellular environment, tissues, and circulation of the host cell (Mohammadzadeh et al. 2020). One of the retained and evolutionary means of secretion is creating extracellular vesicles (EVs) (Mohammadzadeh et al. 2020). EVs are narrowly classified as membrane-bound vesicles that are emitted from cells (Schorey et al. 2015a). Those generated meanwhile in infections may be pathogenic or host-derived (Schorey et al. 2015a). The previous contain gram-negative bacterial outer sheath vesicles and gram-positive bacterial membrane vesicles (Schorey et al. 2015a). These bacteria-generated vesicles' content and construction have recently been below in-depth study and have been well studied elsewhere (Deatherage and Cookson 2012; Kulp and Kuehn 2010; Acevedo et al. 2014). While these vesicles are likely to have a significant role in the generation of extracellular bacterial infection, their function in intracellular infectious pathogens is less evident. The vesicles' transportation mechanisms behind the host cell are not recognized (Deatherage and Cookson 2012; Kulp and Kuehn 2010; Acevedo et al. 2014). Fungal and parasitic pathogens often generate EVs that may serve as immune response modulators (Silverman and Reiner 2011; Oliveira et al. 2013).

Currently, the delivery processes of Mtb proteins to exosomes were found, which proposing that ubiquitination of GroES chaperonin and the HspX protein could transfer them to exosomes (Smith et al. 2015). Additionally, ubiquitination of exosomes’ cargo like Antigen 85 (Ag 85) and ESAT-6 in Human embryonic kidney 293 (HEK-293) cells can prove significant ubiquitination activity transferring proteins to exosomes (Cheng and Schorey 2016). Secretion of EV results in Mtb to liberate their proteins and lipids in a protected route to the recipients, including eukaryotic and prokaryotic cells (Jurkoshek et al. 2016).

Mycobacterium releases EVs in various situations such as culture media, MQ, and the infected lungs in vivo to distribute its crucial components into the extracellular environments (Prados-Rosales et al. 2011). The absence of nutritional components like iron as a natural situation that Mtb faces in the MQs enhance the secretion of EV by Mtb shows that iron accessibility modulates the biogenesis of EV (Prados-Rosales et al. 2014).

EVs of Mtb are generated during iron-restriction conditions and encompass mycobactin, a lipidic siderophore that helps the proliferation of EVs forming Mtb in iron limitation situations (Prados-Rosales et al. 2014). It was demonstrated that the *virR* gene enhances the EVs formation by Mtb, which shows the modulation of vesiculogenesis (Rath et al. 2013). *virR* gene encoding vesiculogenesis and immune response regulator protein that interplays with Rv1488, LpqH, and Rv0383c. In this regard, it seems that these proteins generate a higher-order complex, regulating several aspects of the vesicle formation, such as the EVs size, number, and cargo selection at the cell membrane. It seems that VirR proteins restrict the packing of several immunogenic components in EVs, hence attenuating the Mtb immunostimulatory power and enhances Mtb virulence (Rath et al. 2013). These findings propose a notion that during iron deficiency, the decrease in virR formation increases the secretion of EVs by Mtb (Gupta and Rodriguez 2018). Currently, White and colleagues found the Mtb Pst/SenX3-RegX3 signal transduction system as a new Mtb EV biogenesis regulatory system that works by a unique mechanism compared to VirR protein (White et al. 2018). Current works have found that the mutation of the pstA1 gene in the phosphate-specific transport machinery results in the stimulation of the SenX3-RegX3 two-component system (TCS) and hyper-formation of some proteins like LpqH (an EV-mediated lipoprotein) (White et al. 2018).

Bacterial EVs are the pinches of source bacteria transporting biological substances to distal places in the host, transmitting bacterial virulence genes, and increasing intercellular communication (Pathirana and Kaparakis-Liaskos 2016). The actual process of EV cargo processing has yet to be known. However, it is discovered that certain bacteria have unique machinery for particular packaging cargo inside EVs, while specific other microbial proteins reach EVs based on their charges (Haurat et al. 2011; Elhenawy et al. 2014). Compared to non-pathogenic microbes, pathogens promote more vesicles, confirming the hypothesis that vesicle generation is a standard bacterial system to support bacterial replication and pathogenicity in host cells (Mohammadzadeh et al. 2020). Numerous variables such as temperature, the existence of antibiotics, antimicrobial peptides, serum, and host cell signaling can support microorganisms to manufacture EVs (Mohammadzadeh et al. 2020). The presence of bulky cell walls in Gram-positive microbes and particularly in mycobacteria resulted in a long gap in EV research on these pathogens. The prevailing perception seemed to be that EVs can not be released via the thick cell wall. *Mycobacterium ulcerans* has recently been shown to be capable of developing EVs and even EV biosynthesis in other mycobacterial organisms, including pathogenic species (Mtb) and also non-pathogenic and fast-growing organisms, designating that EV release is a preserved process in the *Mycobacterium* species (Mohammadzadeh et al. 2020).

## Host derived exosome

The phrase host EVs contains exosomes, microsomes, and apoptotic bodies (Witwer et al. 2013; Lötvall et al. 2014). Exosomes are generated from the endosome layer's inward budding as a multivesicular body (MVB) that comprises many exosomes (Bell and Taylor 2017). The MVB then combines with the plasma membrane and leads to discharge exosomes from the cells (Théry et al. 2002; Harding et al. 1984). The generated vesicles can be determined by their antigens, including tetraspanins, CD9, CD63, CD81, and CD82 (Lötvall et al. 2014). Other vesicles in circulating include microsomes formed from plasma membrane destruction larger than exosomes (40–100 nm) 100–1000 nm and apoptotic bodies derived from apoptotic cells with 1–5 μm in scale (György et al. 2011).

The synthesis of exosomes occurs on the cell surface, wherever proteins bounded to the cell membrane could be internalized by endocytosis, which causes the generation of early endosomes (Lässer 2012b). These host proteins may either be pumped back to the plasma layer and degenerate in the endosomal process (Lässer 2012b). Inward vesicles, 40–100 nm in size, are generated by the endosome's internal budding, restraining membrane throughout endosome growth and development (Lässer 2012b). The aggregate of internal vesicles occurs in the production of MVBs (Lässer 2012b). As a couple of evaluations of the membrane have transpired, the membrane introduction of the vesicles created will be the equivalent of the cell's plasma membrane, and their interior will be a part of the cytoplasm (Lässer 2012b). After merging the MVBs with the host plasma membrane, the generated internal vesicles are discharged into the surrounding environment as exosomes (Chaput and Théry 2011; Théry et al. 2009). Throughout the exosomes' construction, there is evidence that the membrane is rearranged because the exosome membrane is reinforced with sphingomyelin and tetraspanins related to the cell plasma membrane (Subra et al. 2007; Kleijmeer et al. 1998).

While the RNA and protein component of exosomes differs and depending on the cell development, it has been discovered that proteins are protected across exosomes of various cellular sources (Mathivanan et al. 2010). These concentrated exosomal proteins comprise cytoskeleton proteins such as ezrin and actin, proteins implicated in MVB biosyntheses such as programmed cell death 6-interactive proteins [alix] and tumor susceptibility gene 101 proteins (TSG101), membrane transportation and fusion proteins such as Rab and annexins, as well as tetraspanins such as CD63, CD9, and CD81 (Mathivanan et al. 2010). Exosomes' role is based on cell development and the cell's present physiological condition because this influences increasing proteins, and RNAs are packed into exosomes (Lässer 2012b). Exosomes originated from different cell sources, and situations have numerous distinct roles (Lässer 2012b). The most well-known roles of exosomes hold their immunostimulatory, immunosuppressive, and tolerance-inducing consequences (Lässer 2012b). These immune-regulatory roles have presented to many clinical investigations in immunotherapy in which exosomes have been employed to enhance the immune response toward pathologic conditions (Chaput and Théry 2011; Chaput et al. 2005).

Exosomes charged with mycobacterial components could activate naïve MQs cells in a proinflammatory route by stimulating the TNF-a, iNOS, RANTES (regulated upon activation, normal T cell expressed, and presumably secreted) formation and exosomes from uninfected cells did not up-regulate these inflammatory markers (Bhatnagar and Schorey 2007; Bhatnagar et al. 2007). In this regard, these exosomes, when exposed to uninfected MQs, trigger a proinflammatory reaction in a Toll-like receptor (TLR)- and myeloid differentiation factor 88 ((MYD88))-dependent manner (Bhatnagar et al. 2007). Besides, exosomes isolated from the bronchoalveolar lavage fluid of *Mycobacterium bovis (M. bovis)* BCG-infected mice contain the mycobacteria components lipoarabinomannan and the 19-kDa lipoprotein and could induce TNF-α production in naive MQs (Bhatnagar et al. 2007). Additionally, exosomes isolated from *M. bovis* BCG- and Mtb-infected MQs, when injected intranasally into mice, induce IL-12 and TNF-α formation as well as neutrophil and MQ recruitment in the lung (Bhatnagar and Schorey 2007; Bhatnagar et al. 2007). Similarly, these exosomes could stimulate CD4 and CD8T lymphocytes, consistent with the production of a powerful acquired immune reaction, and show an alternative process of mycobacterial antigen presentation to these cells instead of the presentation by DCs and MQs (Giri and Schorey 2008). It has been proposed several mechanisms in which exosome could interplay with other host cells. For example, exosomes released from Mtb-infected APCs (antigen-presenting cells) produce MHC-II and present the antigens to T lymphocytes (Ramachandra 2010). Additionally, it has been found that exosomes can carry out whole Mtb proteins such as DnaK, Ag 85 complex proteins, HspX, among others (Giri et al. 2010; Kruh-Garcia et al. 2012). It has been found that the host Hsp70 (heat shock protein 70) is raised particularly in exosomes from Mtb-infected immune cells and is proposed to involve in proinflammatory reactions (Anand et al. 2010). Exosomes liberated from Mtb-infected MQs also inhibit cellular immune reactions mediated to protective immunity, significantly hindering IFNγ-regulated routes that stimulate naïve MQs (Singh et al. 2011). The survival of Mtb inside the host cells mediated to balance between immune reactions like requisite phagocytosis via particular receptors. It is found that exosomes can display a much crucial activity in this regard (Pieters 2008). According to the available evidence, exosomes are potent tools for discovery and design as therapeutic approaches. In this regard, they are simply isolated and purified, diminish the complexity of the bio-fluid source and enrich Mtb proteins, lipids (Kruh-Garcia et al. 2015). Notably, the potential of exosome-based therapeutic biomarkers TB has currently just begun.

## Exosomes isolation and characterization

Several approaches have been proposed to exosomes separation and isolation, and some investigations found exosomes carrying various ingredients that showed numerous activities based on various cell types (Yang et al. 2019). Nevertheless, until now, the purification, isolation, and characterization of exosomes have not been completely defined. Of note, recent investigations concentrated on exosomes as therapeutic markers, drug or gene carriers, and disease markers. As a novel kind of biomaterials with the expansion of biomedical sciences, exosomes have very significant research value; hence, it is crucial to separate exosomes from a wide variety of cells. Various techniques mediated to the size and source of exosomes are proposed to separate exosomes from cell cultures as well as body fluids. In general, five approaches for exosome isolation have been defined, including size-based isolation, ultrahigh-speed centrifugation, immunoaffinity capture, polymer precipitation, as well as microfluidics-derived techniques (Yang et al. 2019). However, the purity of exosomes is a significant issue for the development of exosome-based therapeutics applications (Dauros Singorenko et al. 2017). In this regard, the most usual approach for exosome isolation and separation is ultracentrifugation that is performed for the primary removal of large particles with high sedimentation using low-speed centrifugation. Then, the recovering supernatants go further ultracentrifugation at high speed to earn a pellet containing vesicles.

On the other hand, the characterization of exosomes such as size, protein, and lipid content could be recognized by detecting the particle size, morphology, and surface components on the exosomeʼ surface (Yang et al. 2019). Currently, research on exosomes' function and diagnostic potential is just beginning, and better extraction and isolation of exosomes need further standardization by future investigations. Hence, other sophisticated approaches are needed for the separation, isolation as well as characterization of exosomes. Nevertheless, the selection of best methods for isolation and characterization is needed to enhance the quality of the isolated exosomes and the validity of the findings. In this regard, the main technical challenge in the detection of exosome in therapeutic applications is the differentiation between exosomes collected from normal and pathological cells; that way, it is necessary to design a combination of numerous quantification approaches to discriminate exosome types in heterogeneous samples that can open novel avenues for exosome isolation and characterization.

## Exosome biogenesis

**Fig. 2:**
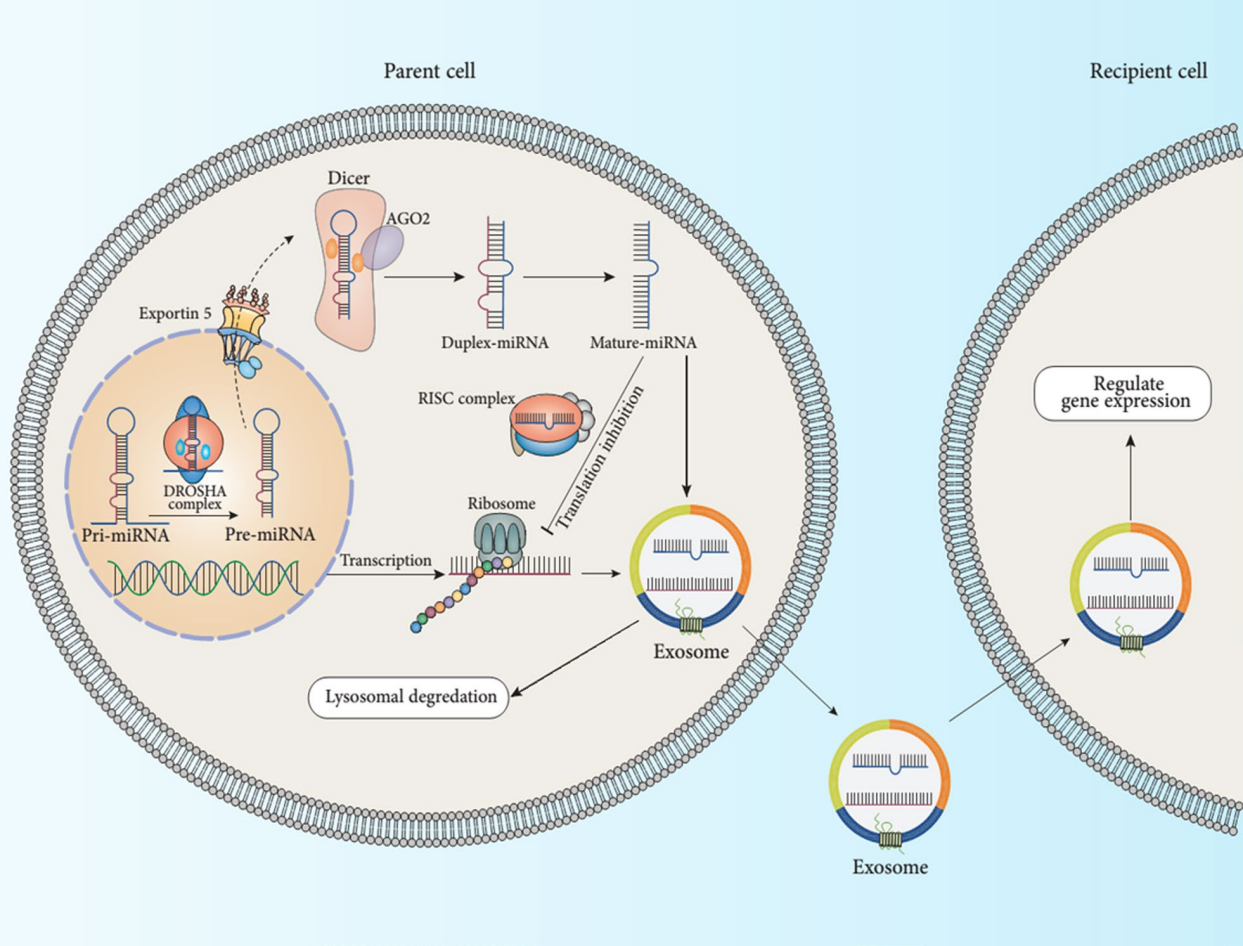
Schematic representation of exosome biogenesis, miRNA internalization, and release. The microRNA or miRNA genes are transcribed into pri-miRNAs (primary miRNAs) in the nucleus. In the next step, pri-miRNAs experiencing a further process by the Drosha complex to form pre-miRNAs (precursor miRNAs). The pre-miRNAs in the Dicer complex were digested to become mature miRNAs. Finally, the mature miRNAs are loaded into exosomes via various possible methods

A significant process for down-regulating and destroying host plasma membrane receptors is their endocytosis and MVB distribution, which could eventually fuse with the lysosome to relate protein degradation (Woodman and Futter 2008; Schorey et al. 2015b; Zhang et al. 2019; McAndrews and Kalluri 2019). A subset of the population of MVBs also combines with the host plasma membrane, culminating in the launch of the intraluminal vesicles (ILV) as exosomes (Woodman and Futter 2008; Schorey et al. 2015b; Yue et al. 2020). Since their characterization, the MVB biogenesis and exosome mechanism is quite being established (Fig. 2) (Théry et al. 2002; Woodman and Futter 2008; Schorey et al. 2015b; Farooqi et al. 2018). Early evaluations in yeast have shown activity for endosomal sorting complexes required for transport (ESCRT) proteins (Hurley 2010). While this ESCRT complex was mainly examined for its activity in the endosomal processing and digestion of ubiquitinated proteins, it effectively reconciled membrane invagination (Davies et al. 2009; Metcalf and Isaacs 2010). Via its ubiquitin-interacting regions, ESCRT-0 clusters have ubiquitinated proteins for transmission to MVBs (Raiborg and Stenmark 2002). ESCRT-0 consequently retains ESCRT-1 to the endosome membrane that, in nature, supplies the surviving components of the ESCRT-III, ESCRT as well as ESCRT-II (Katzmann et al. 2001; Babst et al. 2002). Via the development of polymeric filaments facilitated by ESCRT-III, membrane rupture helps develop ILV (Wollert et al. 2009; Hanson and Cashikar 2012). A series of investigations verify the function of ESCRT organizations in exosome production. The proteomic research of exosomes revealed the existence of ESCRT machines inside exosomes. The elimination of the main components of ESCRT machines could abrogate the development of ILV and the release of exosomes, but this is likely to be cell type-specific (Tamai et al. 2010; Stuffers et al. 2009; Trajkovic et al. 2008). Although this basic concept for MVB biosynthesis has been fully defined, it is not apparent that this is the principal mechanism for the development of MVB. A recent study shows that there are ESCRT-independent pathways for MVB biosynthesis and exosome production. In the oligodendroglial cell line, the exosome development is powered by ceramide's product than by the ESCRT system (Trajkovic et al. 2008). In a study Stuffers et al. (2009) mentioned that the lack of unique subunits from the four ESCRT structures did not entirely prevent the development of MVB. Besides, a process autonomous of both ESCRT and ceramide has been recommended. Work by van Niel et al. (2011) has shown that tetraspanin CD63 exists in high-plenty exosomes, mediates cargo sorting, as well as ILV evolution. Also, CD81 has been shown to interfere with the cargo sorting of tetraspanin ligands like Rac GTPase. However, eliminating these tetraspanins do not resemble to modify the morphology of MVB or the exosomal secretion (Perez-Hernandez et al. 2013). Numerous findings designate that the means for biogenesis of exosome and sorting of protein could be cell-wide or particular to distinct subpopulations of MVBs inside a cell. In this regard, Buschow et al. (2009) have shown that MHC molecules in naive DCs target MVBs that are weak in cholesterol but supplemented with lysobisphosphatide acid that destined for lysosomal degradation. Although MHC molecules in mature DCs are classified into MVBs fortified with CD9 and cholesterol designed for plasma membrane fusion (Buschow et al. 2009).

When MVBs are created, their plasma membrane fusion is driven by the cytoskeleton, fusion machines like SNAP REceptor (SNARE) proteins, and molecular switches like small molecular mass GTPases (Colombo et al. 2014). Rab GTPase is a member of the Ras GTPase superfamily and is recognized to regulate membrane traffickings such as vesicle forming, trafficking, tethering, and fusion with destination cells (Colombo et al. 2014). Approximately 70 separate Rab GTPases have been characterized in mammalian cells to now (Schwartz et al. 2007). Some of these were present on exosomes, specifically Rab11, Rab5, Rab35, and Rab27. These Rab GTPases have been analytically found to act in exosome discharge (Schwartz et al. 2007). Primary examinations showed that Rab11 could be employed to facilitate plasma membrane merging of MVB in the erythroleukemic cell line K562 (Savina et al. 2002). More recent researches have covered Rab35 in interfering plasma membrane docking of MVB in neuroglial cells, where Rab35 deficiency causes a substantial loss of exosome discharge (Hsu et al. 2010). Rab27a, as well as Rab27b, have been observed to have distinguished but usually unnecessary activities in MVB biogenesis, with Rab27a becoming further useful in interceding MVB docking on the plasma membrane (Ostrowski et al. 2010). While Rab GTPases have been involved in the trafficking and merging of MVB, their processing activity is promptly under examination. It is possible to be cell type-based and depend on the host cell (Ostrowski et al. 2010).

## Role of exosome in *Mycobacterium tuberculosis* infection

Exosomes have been shown to bear pathogen-derived ingredients throughout contagious diseases (Beatty et al. 2001; Hassani et al. 2014). Exosomes may serve as immune response modulators regarding their microbial materials package and the critical exposé of pathogenic organisms to the immune system to activate host immune reaction and infection regulation (Hadifar et al. 2019). Furthermore, exposure of pathogenic microorganisms to the immune responses could help transmit pathogens by manipulating immune responses; thus, exosomes may also facilitate disseminating infection (Anand et al. 2010; Schorey et al. 2015b). The modulatory function of exosomes, on the other hand, in the immune reaction is complex and multifactorial (Hadifar et al. 2019). In this regard, it has been found that exosomes originating from Mtb infected MQs cause an inhibitory impact on immune reactions by suppressing IFN-γ‐regulated mechanisms (Singh et al. 2011). On the opposite, some experiments have demonstrated that exosomes originating from infected MQs enhance the production of IFN-γ and TNF-α (Wang et al. 2014). These conflicting results can rely on Mtb Ags availability as exosomal content at every step in the process.

Further research on microbial infections in the sense of exosomes has concentrated on mycobacteria (Bhatnagar and Schorey 2007; Anand et al. 2010; Singh et al. 2015a). Currently, Beatty and Russell (2000) described transportation in mycobacterial ingredients like lipoarabinomannan (LAM) to MVB throughout MQs. They also mentioned that these nanovesicles' material might be present in nearby non-infected MQs (Beatty and Russell 2000). In another study, LAM and 19 kDa proteins were reported as two mycobacterial Ags in separate BAL exosomes of infected mice with *M. bovis* BCG (Bhatnagar et al. 2007). Exosomes' function in TB is now under examination since endocytic trafficking is an essential step in mycobacterial pathophysiology (Singh et al. 2011).

The frontier cells which contact mycobacteria in the lung are neutrophils and MQs (Alvarez-Jiménez et al. 2018). Neutrophils are phagocytes that inhibit bacteria's growth through different methods, including lysosomal enzymes and antimicrobial molecules found in one's lysosomes and the release of ROS (Alvarez-Jiménez et al. 2018). These phagocytes often discharge EVs into the extracellular environment; these EVs comprise a lipid bilayer covering the hydrophilic center and engaging in intercellular communication (Alvarez-Jiménez et al. 2018). In vitro infected human neutrophils with Mtb cause to produce EVs; however, these EVs' impacts on another cell are critical to the regulation of Mtb disease mechanism, such as MQs not been thoroughly studied (Table 1) (Alvarez-Jiménez et al. 2018). In an investigation by Alvarez-Jiménez et al. (2018), Four types of EVs were identified, including EVs formed by human neutrophils (non-stimulated) (EV-NS), EVs generated by activator-stimulated neutrophils (PMA), EVs produced via a peptide obtained from bacterial proteins (f MLF) or Mtb, which varied in their stability. In EV-TB, TLR ligands 2/6 were observed. These EVs supported a moderate upregulation of CD80 co-stimulatory molecules, greater levels of CD86, and the generation of immense levels of TNF-α, as well as IL-6, and lesser concentrations of transforming growth factor-β (TGF-β) in allogeneic human MQs relative to other EVs (Alvarez-Jiménez et al. 2018). EV released from TB decreased the volume of intracellular Mtb in infected MQs and enhanced superoxide. TLR2 and 6 ligation and superoxide anion generation are recognized autophagy activators; thus, Alvarez-Jiménez et al. (2018) observed that EV-TB stimulated increased abundance of the LC3-II autophagy indicator in MQs and co-location of LC3-II with Mtb in infected MQs. In these cells, a load of intracellular mycobacterial boosted was autophagy blocked by wortmannin (Alvarez-Jiménez et al. 2018). In conclusion, the findings of Alvarez-Jiménez et al. (2018) showed that neutrophils generate numerous EVs in reply to various actuators and that EV discharged from TB infected cells excites MQs and facilitates the removal of intracellular Mtb by initial stages of superoxide anion production and autophagy induction, an innovative function for EVs-derived from neutrophil the immune reaction to Mtb.

**Table 1:** Exosomes (vesicles) derived from Mycobacterium and host-derived (macrophages and neutrophils)

Mycobacterium	Macrophages/Neutrophils
The vesicles derived from mycobacterium were first visualized by scanning electron microscopy (SEM). These vesicles were discovered in the extracellular matrix of *Mycobacterium ulcerans* biofilm and from biopsies of Buruli ulcer-like lesions in infected mice. Also, these vesicles carried the sole virulence factor accountable for Bureli ulcer, the lipid toxin mycolactone, and, accordingly, presented potent cytotoxic activity (Marsollier et al. 2007; George et al. 1999)	The exosomes originate from macrophages infected with *Mycobacterium avium* carry diverse antigens of both *M. avium* and the host cell. In macrophages, they are implicated in the initiation and creation of inflammatory responses. The interplay among the exosomes derived from macrophages infected with *M. avium* and phagocytosis (macrophage), cytokine production, immunostimulation, and apoptosis was investigated. In macrophages treated with exosomes (derived from macrophages infected with *M. avium*), the phagocytosis of dextran by macrophages was enhanced. Besides, the expression of CD40, CD80, CD81, CD86, Human Leukocyte Antigen (HLA)-DR, and most notably, CD195 was improved. Moreover, Interleukin (IL)-6, IL-8, IL-10, IFN-γ, and tumour necrosis factor α (TNFα) were increased by stimulated macrophages (Wang et al. 2015)
The investigation results showed that the extensive cell envelope restructuring associated with vesicle discharge correlated with modulation of cell surface lipid biosynthesis and peptidoglycan remodelings. Comparative transcriptomics explained common high expression of the iniBAC operon associated with high vesicle generation in *Mycobacterium tuberculosis* (Mtb) cells. Vesicle generation examination demonstrated that the dynamin-like proteins (DLPs) encoded by this operon, IniA, and IniC, are required to release extracellular vesicles (EVs) by Mtb in culture and infected macrophages (Gupta et al. 2020)	EVs were originated from J774A.1 macrophages infected with Mtb H37Rv varied in size and phosphatidylserine content from directly discharged EVs. These EVs additionally had distinct physiological impacts: S-EV diminished the mycobacterial load and cytokine generation in vitro (through a phosphatidylserine-dependent mechanism). In contrast, both EVs decreased the bacterial lung load in vivo. These results are the foundation for more investigations to assess whether EVs enhance the efficacy of the conventional therapy for tuberculosis (TB) (García-Martínez et al. 2019)
The recent evidence has indicated that the Mtb infection can enhance microvesicle generation in response to iron limitation. These microvesicles carry mycobactin, which can work as an iron donor and promotes the replication of iron-starved mycobacteria. Finally, the study results revealed a function of microvesicles in iron attainment in Mtb, which can be significant for durability in the host (Prados-Rosales et al. 2014)	The EVs derived from macrophages infected with *M. bovis* BCG are carriers of mycobacterial cell wall lipids such as lipoarabinomannan (LAM) and phosphatidylinositol mannoside (PIM). These molecules provoke the production of chemokines and cytokines, which leads to inflammation. The activation of nuclear factor-κB (NF-κB) is associated with the intracellular endurance of mycobacteria. In fact, the mycobacterial proteins Rv2456c, MPT64, PPE37, and Rv3402c activate NF-κB (Wang et al. 2019)
A study uncovered EVs were provided with pleasant quality composition with intact conformational construction during the isolation procedure. The isolated EVs had the initial qualifications as an immunogenic particle, such as safety, perseverance, inexpensiveness, and antigens possession, which, based on the relationships between *Mycobacterium kansasii* and Mtb, make them a proper competitor for prospective prophylactics, curative, detection, and adjuvants investigations against mycobacterial pulmonary diseases (Hoseini Tavassol et al. 2017)	When exposed to uninfected macrophages, exosomes stimulate a proinflammatory response in a Toll-like receptor—and myeloid differentiation factor 88—dependent fashion. Besides, exosomes separated from the bronchoalveolar lavage fluid (BALF) of mice infected with *M. bovis* BCG carry the mycobacteria components lipoarabinomannan and the 19-kDa lipoprotein. They can incite TNF-α generation in naive macrophages. Moreover, exosomes separated from *M. bovis* BCG- and Mtb-infected macrophages, when injected intranasally into mice, stimulate TNF-α and IL-12 production and neutrophil and macrophage recruitment in the lung. These investigations recognize an earlier undiscovered role for exosomes in developing intercellular signaling throughout an immune response to intracellular pathogens. They hypothesize that the extracellular release of exosomes carrying pathogen-associated molecular patterns (PAMPs) is a crucial immune surveillance mechanism (Bhatnagar et al. 2007)
EVs were also seen in macrophages and mice infected with Mtb and *Mycobacterium bovis* Bacille Calmette-Guérin (BCG)-mice. Recently, EVs of bacterial sources were separated from the tissue culture medium of macrophages infected with Mtb, designating that Mycobacterial extracellular vesicles (MEVs) are discharged into the extracellular milieu throughout intracellular infection. However, the molecular mechanisms implicated in MEVs trafficking out of the phagosome and through the macrophage plasma membrane are unexplained (Gupta and Rodriguez 2018)	A study displayed that Mtb RNA is transported into EVs derived from macrophages through an Mtb SecA2‐dependent pathway. EVs released from macrophages infected with Mtb can incite a host RIG-I (Retinoic acid-inducible gene I)/Mitochondrial antiviral-signaling protein (MAVS)/TANK-binding kinase 1 (TBK1)/IFN regulatory factor 3 (IRF3) RNA sensing pathway, driving to type I interferon generation in receiver cells. In a RIG‐I/MAVS‐dependent manner, these EVs also promote the maturation of Mtb‐containing phagosomes through a noncanonical LC3 pathway, pointing to enhanced bacterial removal (Cheng and Schorey 2019)
A report revealed that the two medically essential species of mycobacteria, Mtb, and *M. bovis* BCG, release Mycobacterial vesicles (MVs) during growth in in-vitro and in-vivo conditions (both liquid culture and inside the murine phagocytic cells). They documented MV generation in different virulent and nonvirulent mycobacterial species, designating that the production of MVs is a characteristic conserved amongst mycobacterial species. Comprehensive proteomic investigation revealed that only MVs from the virulent strains carried Toll-like receptor (TLR)-2 lipoprotein agonists. The interplay of MVs with macrophages separated from mice stimulated the generation of cytokines and chemokines in a TLR2-dependent mode. The infusion of MVs toward mouse lungs evoked a florid inflammatory reaction in WT but not TLR2-deficient mice (Baena et al.)	It has been designated that the several EVs generated by non-stimulated human neutrophils (EV-NS), EVs created by neutrophils incited with an activator (PMA), a peptide derived from bacterial proteins (fMLF) or Mtb; have differed in their size. In TB-EVs, the ligands for toll-like receptor (TLR) 2/6 were detected. These EVs promoted a moderate rise in the production of the co-stimulatory molecules CD80, a greater expression of CD86, higher volumes of TNF-α and IL-6, and lower masses of transforming growth factor beta (TGF-β), in autologous human macrophages, contrasted with the other EVs (Alvarez-Jiménez et al. 2018)
MVs released by Mtb carry lipoprotein LpqH, a central agonist for host TLR2. This study identifies a gene, rv0431, which appears to regulate mycobacterial MV formation, and therefore we suggest it be named “vasculogenesis and immune response regulator” (virR). This gene encodes a protein that holds a unique fold, as defined by nuclear magnetic resonance (NMR) spectroscopy, and a disordered domain indicative of cooperation in a higher-order network. By limiting the discharge of most of the material delivered by Mtb that stimulates host cells by TLR2, VirR declines Mtb’s immunostimulatory potential and enhances its virulence (Rath et al. 2013)	Gonzalez-Cano et al. (2010) reported the exosomes generated from neutrophils infected with Mtb H37Rv, carried CD35, Rab5, Rab7, gp91phox, phosphatidylserine, and enzymes such as myeloperoxidase and elastase. This research combines another cell of the innate immune system to the representation of EVs as alleged effectors

Endothelial cells play a significant role in modulating immune reaction by promoting leukocyte permeability and cell movement and developing cytokines, including TNF-α (Li et al. 2018). Control of endothelial cell immune activity is strictly regulated, and novel verdicts designate that EVs play a significant part in this mechanism (Li et al. 2018). However, EVs' function in controlling endothelial activation in the sense of pathogenic bacteria is overlooked (Li et al. 2018). Li et al. (Giri and Schorey 2008) identified the endothelial cell reaction to EVs derived from MQs infected with Mtb to start tackling this information gap to begin addressing this knowledge gap. The outcome shows enhanced MQ mobility through the monolayer when endothelial cells were pretreated with EVs isolated from MQs infected with Mtb (Li et al. 2018). The transcriptome investigation revealed a substantial upregulation of the genes implicated in cell adhesion and inflammatory means in endothelial cells managed with EVs (Li et al. 2018). Pathway examination of these preferentially expressing genes revealed that many immune response-related processes had been upregulated (Li et al. 2018). Endothelial cells have also been treated with EVs derived from the serum of mice infected with Mtb and, curiously, EVs obtained 14 days but not 7 or 21 days after infection displayed the similar capacity to provoke endothelial cell activation, designating a variation in EV activity during Mtb infection course (Li et al. 2018). The immunofluorescence microscopy result showed that NF-κB and Type1 IFN pathways were implicated in endothelial stimulation by EVs. In sum, Li et al. (2018) found that EVs may trigger endothelial cells and play a crucial role in modifying host immune function throughout Mtb infection.

## Host derived microRNAs

MiRNAs are small non-coding RNAs with a length of about 18–22 nt that contribute to many fundamental cellular processes (Zhang et al. 2015). The roles of miRNAs in various approaches have been fully established, such as cell growth, cell differentiation, cell immigration, infection onset, and disease progress (Zhang et al. 2015; Png et al. 2012; Gee et al. 2008; Tay et al. 2008). Some miRNA genes are carried inside protein-encoding introns and also non-coding genes (Janas et al. 2011). The bulk of miRNAs are deciphered to RNA polymerase II as long primary miRNAs (Nahand et al. 2020; Mirzaei et al. 2020a,b). Besides, cyclin-dependent kinases such as CDKF1 and CDKD phosphorylate the C-terminal region of the most significant RNA polymerase II subunit (Achkar et al. 2016). Some transcription agents regulate the transcription of miRNA genes and miRNA-related pathways; for instance, NOT2 communicates with RNA polymerase II and manages the transcription of some miRNA genes (Achkar et al. 2016). Primary miRNA (pri-miRNA) comprises a hairpin system consisting of a terminal circle and a stem area (Nahand et al. 2020). Pri-miRNA is 5-coated, spliced, and polyadenylated, creating a single stable miRNA molecule (Nahand et al. 2020; Siomi and Siomi 2010). RNase III enzymes, Dicer, and Drosha directly process these pri-miRNAs to produce mature duplex miRNAs comprising 18–22 nucleotides (Meijer et al. 2014). In the nucleus, 'microprocessor,' a multi-protein network, pri-miRNAs are first grown into a nearly 70 nucleotides hairpin-structured substrate named pre-miRNA (Nahand et al. 2020). Next, Drosha and DGCR8/Pasha (which is the dsRNA-binding region [dsRBD] protein), which have been the two main parts of the microprocessor, along with the cofactors DDX5, p72 (DDX17), and heterogeneous nuclear ribonucleoproteins (hnRNPs) function together to connect the double-stranded area of the pre-miRNA to create a 2-nt 3 overhang that is recognized by XPO5 and facilitates the translocation of the pre-miRNA (Nahand et al. 2020; Kim et al. 2009; Okada et al. 2009). In the cytoplasm, Dicer (a different RNase III enzyme) working with the dsRBD protein, TRBP/PACT, additional splitting the pre-miRNA to a nearly 22-nt extended miRNA duplex (Nahand et al. 2020). One strand of miRNA duplex, commonly A/G rich strand with a 5-U initiating site called a guide strand, attaches to Argonaut (AGO) protein inside the RNA-induced silencing complex (RISC) (Nahand et al. 2020). The duplex passenger strand is rich in U/C and typically starts at 5-C and is doomed to have deteriorated. Dependent on expression profiling, the two strands may be similarly dominant in specific tissues (Meijer et al. 2014). The miRNA regulates the RISC to attach the target mRNA element to the 3 UTR, thereby destroying the mRNA, providing to the deregulation of the target gene expression (Siomi and Siomi 2010; He and Hannon 2004). Some alternative Drosha-independent miRNA means have been discovered, such as tRNA-and snoRNA-derived miRNAs and mirtrons (Siomi and Siomi 2010; He and Hannon 2004). While one strand of miRNA is packed into Ago for mRNA linking leading to gene repression, through RNA-binding proteins (RBPs), the other strand is transferred to the plasma membrane for discharge in microvesicles or to MVBs for release in exosomes (Nahand et al. 2020). The evaluation of the miRNA quality of exosomes obtained from cardiac fibroblasts exhibited the abundance of many miRNA passenger strands (Bang et al. 2014). The loss of Ago2 from exosomes implies that exosomal miRNAs are handled by RBPs and protected against degeneration (Koppers-Lalic et al. 2014). RBPs perform a part in carrying miRNA strands to MVBs for packing onto exosomes or to the plasma excretion membrane (Nahand et al. 2020). Subsequent conveying endosomes into the trans-Golgi network, they can be delivered to declining lysosomes or combine with microtubules to connect to the plasma membrane, providing to the release of ILVs as exosomes into extracellular place (Nahand et al. 2020). Exosomes may communicate with receiver cells via membrane merging, endocytosis, or juxtacrine signaling (Nahand et al. 2020).

## Role of host miRNAs in *Mycobacterium tuberculosis* infection

Mtb is an archaic pathogen that has been correlated with hosts such as a human. Hence, it has been adjusted to the host cell's phagocytes for survival (Saltini 2006). Until now, very little is recognized on how the MQ response evolves throughout TB infection by human miRNAs, which would be the initial phagocyte immune reaction in the respiratory microenvironment attributable to the Mtb pathogen (Saltini 2006). Bacterial pathogens exploit a wide variety of host cell processes and functions to ensure the preservation and spread (Bhavsar et al. 2007). Modulation of miRNA expression by disease because of bacterial pathogens in infection befalls is required for the host response to an infection and a novel biological strategy for modulating host cell mechanisms by bacteria (Bhavsar et al. 2007). MQs are the central target cells for infection with Mtb are not influenced by miRNAs throughout infection (Bhavsar et al. 2007). The endogenous and adaptive immune response's key point is DCs that could prompt and polarize the T cell activation controlled by miRNAs (Mehta and Liu 2014). MiRNAs play a crucial function in maintaining MQ's primary activity, DCs, and NKCs (Bezman et al. 2010; O'Connell et al. 2010). Many experiments have demonstrated a shift in the expression of the genes in MQs and NKC due to dormant and active TB and healthy people, relative to those with TB (Behrouzi et al. 2019). MiRNAs control alterations in gene expression and variance in cell structure, and some miRNAs regulate the differentiation of T cells and their function (Lui et al. 2014). Ni et al. (2014) have shown that the intrinsic MQ activation mechanism could alter multiple miRNAs' control. They also found the Mtb changes some host miRNAs such as miR132, and miR-26a, attenuating the immune reaction to maintain stability (these miRNAs usually serve as negative MQ role controllers through IFN-γ) (Ni et al. 2014). In the scenario of pulmonary TB, the initiation of mentioned miRNAs into alveolar MQs restricts the immune reaction and deteriorates the alveolar area (Ni et al. 2014). Previous experiments, on the other hand, have explained that miR-361-5p is slightly close to the level of bleomycin-provoked fibrosis in the lung of mouse and can be implicated in identifying the causes of lung injury, as well as fibrosis (Xie et al. 2011). Fu et al. (2011) have demonstrated for the first time that raised masses of miR-361 have been produced in the serum of cases with TB related to healthy people, and it can be hypothesized that this represents lung damage owing to TB infection, although the related process remains unknown.

## Sorting of microRNAs into exosomes (exosomal RNAs)

A significant key point in the area of exosomes emerged in the current decade. It was shown that human exosomes from mast cells comprised more than 1200 messenger RNA (mRNA) copies that might be transported to other cells would be turned into proteins (Valadi et al. 2007). Besides, miRNA was discovered in these exosomes. Since it was previously proposed that one miRNA might influence the translation of 100–200 mRNAs, this offered more evidence for exosomes' fundamental function in cell-to-cell interaction (Lässer 2012a). The discovery of RNA in exosomes advanced novel visions into exosomes' roles and recommended the potential application of exosomes as biomarkers for disorders or applied as vectors in gene therapy. Increasing data have demonstrated that miRNAs are abundant in body fluids like saliva, vomit, breast milk, and blood (Gallo et al. 2012; Michael et al. 2010; Lv et al. 2013; Zhou et al. 2012; Hu et al. 2010; Arroyo et al. 2011). Extracellular miRNAs and being loaded into exosomes or microvesicles may be inserted into high-density lipoprotein (HDL) or attached to the AGO2 protein out of the vesicles (Arroyo et al. 2011; Tabet et al. 2014; Vickers et al. 2011). Both three forms of action guard miRNAs from deterioration and maintain their durability. As mentioned above, exosomes include an extensive range of molecules, such as proteins, lipids, DNA, mRNAs, and miRNAs, which are described in the ExoCarta database (Simpson et al. 2012). MiRNAs have obtained the most engagement in these molecules owing to their regulating functions in gene expression. Goldie et al. (2014) have shown that the percentage of miRNA in exosomes between small RNAs is more extensive than in their progenitor cells.

MiRNAs are not automatically inserted into exosomes, as defined profiling investigations have confirmed. Guduric-Fuchs et al. (2012) thought about the amounts of miRNA expression in several cell lines and their associated exosomes. They recognized that a subclass of miRNAs such as miR-142-3p, miR-150, as well as miR-451 ideally reached exosomes. Besides, several investigations have also found that exosomal miRNA rates are changed below different physiological circumstances. MiR-21 was reduced in serum exosomes of healthy subjects than in cases with glioblastoma (Skog et al. 2008). In plasma vesicles of non-small cell lung carcinoma patients, miR-20b, let-7f, and miR-30e-3p are lower than standard controls (Silva et al. 2011). These findings indicate that the parent cells have a sorting system that directs unique intracellular miRNAs into exosomes.

Experiments have shown that there is a subset of miRNAs that are spontaneously categorized into exosomes, including miR-150 and miR-320 (Zhang et al. 2015). The miR-320 members are commonly spread in exosomes originating from natural tissues and tumors (Guduric-Fuchs et al. 2012; Skog et al. 2008; Liao et al. 2014; Squadrito et al. 2014; Huang et al. 2013). MiR-150 is strongly generated in HEK 293T-derived exosomes, peripheral blood of tumor individuals, colony-stimulating factor 1 (CSF-1)-derived MQs of bone marrow as the serum of colon cancer cases (Zhang et al. 2015). Also, distinct miRNAs, for instance, miR-451, are widely expressed in exosomes originating from healthy cells like HEK-293T cell line, HMC-1 cell line, primary T lymphocytes Epstein–Barr virus-transformed B-cell lymphocytes (Zhang et al. 2015). Other miRNAs like miR-155 and miR-214 are supplied with exosomes created from tumor cell lines and peripheral blood from people with cancer (Zhang et al. 2015). Based on existing studies, there are plausible methods for sorting miRNAs onto exosomes, but the fundamental mechanisms stay widely unknown. These include a neuronal pathway based on sphingomyelinase 2 (nSMase2). NSMase2 is the primary molecule designated to be connected to miRNA discharge in exosomes (Zhang et al. 2015). Kosaka et al. (2013) remarked that overexpression of nSMase2 boosted the rate of exosomal miRNAs and, contrarily, suppressed the expression of nSMase2 decreased the rate of exosomal miRNAs. B, The sequence of miRNA and heterogeneous nuclear ribonucleoproteins (hnRNPs)-dependent route (Kosaka et al. 2013). Villarroya-Beltri et al. (2013) recognized that the hnRNPA2B1 could distinguish the GGAG pattern in the 3′ component of the miRNA sequences and allow unique miRNAs to be loaded into exosomes. Other hnRNP family proteins such as hnRNPA1 and hnRNPC could also attach to exosomal miRNAs, designating that they may also be applicants for miRNA sorting. Nevertheless, no binding explanations have yet been established (Villarroya-Beltri et al. 2013). C, The 3′-end of the miRNA sequence-dependent route. Koppers-Lalic et al. (2014) recognized that 3′ ends of uridylated endogenous miRNAs were chiefly present in exosomes originating from B cells and urine. In contradiction, 3′ ends adenylated endogenous miRNAs were mainly present in B cells. The above two filtering modes ordinarily intend that the 3′ or 3′ end of the miRNA series comprises a fundamental sorting signal. D, The mechanism connected to the miRNA-mediated silencing complex (miRISC). It is fully confirmed that mature miRNAs can correlate with assembly proteins to produce a complex termed miRISC. MiRISC's key elements contain AGO2, miRNA, miRNA-repressible mRNA, as well as GW182. The AGO2 protein performs a significant function in mRNA mediation: the evolution of miRNA and the following translational abolition deterioration mRNA molecule (Frank et al. 2010). Latest investigations have verified a possible connection between AGO2 and the sorting of exosomal miRNA. Guduric-Fuchs et al. (2012) remarked that AGO2 knockouts could depreciate the types and rates of preferentially transported miRNAs like miR-150, miR-451, as well as miR-142-3p, in HEK 293T-derived exosomes. Further study also reinforced the interaction between sorting of exosomal miRNA and miRISC. Second, the key elements of miRISC were found to be co-located with MVBs (Zhang et al. 2015). Second, hindering the turnover of MVBs in lysosomes might contribute to the over-accumulation of miRISCs, while preventing the expansion of MVBs led to the loss of miRISCs (Zhang et al. 2015). Third, alterations in miRNA-repressible target levels that befall in reply to cell activation can result in sorting of miRNA to exosomes, partially by differential interference at miRNA action sites (miRISCs) and exosome biogenesis sites (Zhang et al. 2015). In sum, some miRNAs' unique sequences can direct their integration into exosomes. However, certain enzymes or other proteins can also regulate the triage of exosomal miRNAs independently of the miRNA sequence.

## Exosomes and immune responses in tuberculosis

Exosomes often contain Ags from the cells where they come. These Ags can be modified to peptide following exosome capturing by DCs and paired with MHC molecules for eventual T cells presentation (Bobrie et al. 2011). For example, exosomes secreted by cells infected with a pathogen, such as Mtb or *M. bovis*-infected MQs, as well as cytomegalovirus-infected endothelial cells, carry microbe Ags and facilitate the activation of pathogen-specific CD4 and CD8 T-lymphocyte reactions (Bhatnagar and Schorey 2007; Walker et al. 2009).

While intracellular miRNAs are identified as the leading players in the control of gene expression in eukaryotic cells, particularly in immune cells, extracellular-released miRNAs' physiological role has only opened to be investigated (He and Hannon 2004; Xiao and Rajewsky 2009; Bronevetsky and Ansel 2013). Valadiet al. (2007) was the initial to illustrate that exosomes comprise messengers and regulatory RNAs (such as miRNAs) in their membrane compositions. Simultaneously, not DNA nor ribosomal RNA (18S and 28S rRNA) is recognizable. Also, vesicle transport was characterized as unique, presented that mast cell-derived EVs were internalized by specific mast cells but not through CD4 T cells (Valadi et al. 2007). After the preliminary examination, RNA and miRNAs have been presumably recognized in vesicles generated by most of the cells experimented with stem cells to neurons, blood, hepatocytes cells (Candia et al. 2016). Much focus has been given to pathological statuses, but some latest discoveries have also explained the physiological importance of miRNA release. Instances of miRNAs in EVs as a cell–cell signaling pathway are hematopoietic modulation in the bone marrow, muscle cell development, and crosstalk among astrocytes and neurons (Salvucci et al. 2012; Forterre et al. 2014; Morel et al. 2013).

In inflammatory environments, matured MQs develop and discharge EVs at massive levels that can cause phenotypic segregation and monocytes' functional development in other MQs (Candia et al. 2016). Notably, these EVs are supplemented with miR-223, which would be essential for vesicle-induced monocyte development. Therefore, they are members of a feedback loop that distinguishes between the hired monocytes and generates further vesicles as a local reaction that stimulates the native immune system (Ismail et al. 2013). Throughout the development of immune synapses, T-cell-released EVs comprise miRNAs passed to APCs in a unidirectional and antigen-driven manner and can attenuate gene expression in receiver cells (Mittelbrunn et al. 2011; Gutiérrez-Vázquez et al. 2013). There are key characteristics of this way of communicating: (1) the vesicle range of miRNA varies significantly from that of parental cells, intimating that the collection of released miRNAs could be strongly controlled; and (2) the correlation of cognate cells produced by immune synapses is required to facilitate the secrete of vesicles on one side and to cause fusion (Mittelbrunn et al. 2011; Gutiérrez-Vázquez et al. 2013). MiRNAs correlated with EV were also determined to be distinct in mature and immature DCs and to engage effectively in these cells' intermodulation to rebalance APCs and the immune response (Montecalvo et al. 2012). DCs interact via vesicle interchange with distinct miRNAs having distinct and unique physiological functions: of note, EV-delivered miR-155 increases while miR-146a lessens inflammatory responses, intercedes target gene suppression, and reprograms the cellular reaction to endotoxin (Alexander et al. 2015). This cell contact mechanism is envisaged to happen in a paracrine situation, with vesicles moving very slight distances or even transporting from one cell to another by direct cell–cell contact. Nevertheless, EV-mediated miRNAs could often transfer distances and become endocrine signals (Candia et al. 2013).

MQs as the crucial line for defense toward Mtb can present Mtb antigens via exosomes (Wang et al. 2018). Mtb following invading to the host cells can be lysed via several hydrolyze mechanisms in MQs, and Mtb parts are degraded into short-peptide components (Wang et al. 2018). The complex produced via short-peptides attaching to MHC-I/II on the surface of host exosomes and could be transferred to the surface of MQs to stimulate MQs and CD4 and CD8 T cells, thereby forming a particular immune reaction toward Mtb infection (Singh et al. 2015a; Raposo et al. 1996). Additionally, the enhanced inflammatory chemokines like IFN-γ, IFN-α, IL-12, and TNF-α would be stimulated significantly to increase the inflammatory reactions by host cells (Cheng and Schorey 2013). In this regard, exosomes carrying these proteins trigger the growth and stimulation of T cells and the TNF-α formation to increase cellular immune activities (Wang et al. 2015). Exosomes generated from Mtb-infected MQs with several parts of Mtb like the mannosylated LAM, which transfers immunologic information among MQs and impacts the TNF-α formation to kill Mtb (Bhatnagar et al. 2007).

Additionally, exosomes carrying LAM can impact the signaling and response of T lymphocytes (Schorey and Harding 2016). Exosomes generated from Mtb-infected MQs can stimulate CD8 and CD4 T cells, such as the stimulation and maturation of DCs, and stimulate the inflammatory reactions by IL-8 and IL-6 to kill Mtb (Giri and Schorey 2008; Wang et al. 2015; Mahon et al. 2012). Besides, exosomes can partially suppress TLR-2 and MYD88 formation, suppressing the MQs to IFN-γ (Singh et al. 2011; Harding and Boom 2010). Exosomes, in host cells, mediated to E1, E2, and E3 ubiquitin ligases to deliver the Mtb components among host cells, show ubiquitination can act as the factor for bacterial infection (Smith et al. 2015; Schorey et al. 2015b). These findings show exosomes generated by host cells carrying proteins of Mtb that can stimulate specific and non-specific immune reactions in host cells to eliminate Mtb. Kruh-Garcia et al. (2014), in a study by Selected reaction monitoring (SRM)-mass spectrometry, showed that more than 20 types of Mtb proteins such as Ag85, bfrb, Apa, glcB, katG, HspX, as well as MPT64 carrying exosomes from of TB individuals. Besides, HSPs like HSP60, HSP70, and HSP-90 in exosomes from LTBI individuals were up-regulated significantly. These proteins can be recognized quickly, showing they can be administrated as therapeutic biomarkers for quick diagnosis of LTBI, offering the powerful diagnostic approaches for monitoring Mtb infection in people with high-risk exposure and advanced TB (Shekhawat et al. 2016).

The exosomes generated from Mtb-infected MQs could interplay with host immune cells and induce the formation of proinflammatory mediators by MQ like TNF-α and stimulation of naïve antigen-specific T lymphocytes (Singh et al. 2011). Nevertheless, exosomes carrying Mtb parts can also regulate MQs activity to enhance the survival of Mtb. In this regard, one possible mechanism is the capability for exosomes to render MQs refractory to subsequent stimulation by IFN-γ.

Additionally, it has been shown that *M. bovis* BCG-derived exosomes from infected MQs could induce growth and IFN-γ formation by pre-sensitized CD8 and CD4 T lymphocytes (Giri and Schorey 2008). The bacterial antigens encounter in this re-stimulation should be characterized. However, some factors, such as Ag85 (highly immunogenic factor), are one candidate, and also, lipids from bacterium on exosomes can induce a T lymphocyte reaction via CD1 (Giri and Schorey 2008). It has been found that re-stimulation of T lymphocytes by exosomes was significantly increased in the presence of APCs, proposing that the function of exosomes is high in cross-priming in direct stimulation of T lymphocytes. A study by Giri and Schorey (2008) found that intranasal apply of exosomes from *M. bovis* BCG-infected MQs can stimulate naïve T lymphocytes and that these T lymphocytes respond with IFN-γ formation upon re-stimulation with *M. bovis* BCG antigens. Besides, these exosomes stimulated the development of powerful memory CD8 and CD4 T lymphocytes (Giri and Schorey 2008). Hence, these finding show that exosomes generated from *M. bovis* BCG-infected MQs can act as a practice preventive strategy. However, further investigations are needed to dissect the phenotype of the T lymphocytes stimulated after exosome administration and, most notably, determine if intranasal use of exosomes from *M. bovis* BCG and Mtb -infected MQs and as well as DCs can protective toward Mtb infection in individuals.

## Exosomal RNAs in *Mycobacterium tuberculosis* infection

The TB eradication has partially restrained due to Mtb's potential capacity for continues dormant in the host (human) over time without triggering the disease, a condition held to as latent (Velayati et al. 2016; Alipoor et al. 2016a). A systematic proteomic investigation has been established to determine exosome content proteins in infected MQs with dead or live Mtb in in-vitro (Giri et al. 2010). This defined the prevailing function of host proteins and 41 mycobacterial proteins in discharged exosomes (Giri et al. 2010). Much investigation showed the presence of SAT-6 Ag (Rv3875), MPT64, Ag85 complex, and MPT63, which are strongly immunogenic Mycobacterium proteins (Anand et al. 2010; Kruh-Garcia et al. 2015; Booton and Lindsay 2014). Following investigation established 20 mycobacterial proteins in serum-isolated exosomes in subjects with TB, like Ags GlcB, 85b, BfrB, as well as Mpt64 (Kruh-Garcia et al. 2014). It is also conceivable to distinguish respiratory and extra-respiratory TB from exosomal serum markers like MPT64 and identify latent and active conditions (Kruh-Garcia et al. 2014). The detection of latent capacity from active disease is significant in the endemic community and may enhance at-risk individuals' screening and deter infection spread. A similar Mtb outcome in exosomes exists in human specimens, cell culture, and animal models (Kruh-Garcia et al. 2015; Ardekani and Naeini 2010). Exosomes from designed culture filtered proteins (CFP)-treated MQs can trigger immune responses (innate and adaptive) (Alipoor et al. 2016a). In such exosomes, 29 Mtb proteins have been recognized, with the bulk contrasted with those detected in exosomes obtained from Mtb infected-MQ (Alipoor et al. 2016a). These exosomes might activate MQs, DCs, as well as naive T lymphocytes in vivo (Alipoor et al. 2016a). These findings imply that EVs such as exosomes containing Mtb Ag's cargo can produce TB vaccines reliant on CFP (Alipoor et al. 2016a; Booton and Lindsay 2014). Exosomes are often documented to serve as carriers of Pathogen-associated molecular patterns (PAMPs) and affect the receiver cells by either suppressing or stimulating immune function (Schorey et al. 2015b). Mtb can cause partial protection to IFN-γ activation in infected MQs through PAMPs, including 19 kDa lipoprotein and mycolylarabinogalactan-peptidoglycan composite coupling to TLR-2 display on MQs (Fortune et al. 2004). Exosomes generated by MQs infected with Mtb imitate this impact (Singh et al. 2011). According to the genome-wide findings on Mtb disease, it has caused a miRNA formation profile in primary human MQs (Furci et al. 2013). After infection, MQs with avirulent *M. Bovis* *BCG *and Mtb of H37Rv (virulent) miRNA expression patterns mainly alternating among the two live mycobacteria studied. However, a significantly distinct model evolved from the infection by dead Mtb, indicating a dynamic miRNA metabolism affected by live intracellular pathogens (Furci et al. 2013). Taken together, the level of Mtb exosomal miRNAs in MQs (infected with Mtb) is slightly smaller relative to non-infected cells. More than 100 mRNAs have been specific to infected cell exosomes and could be active in modulating recipient cells' immune reactions (Singh et al. 2015a). This evidence helps the functional and diagnosis capacity of exosomal mRNAs, as well as miRNAs in TB.

**Fig. 3:**
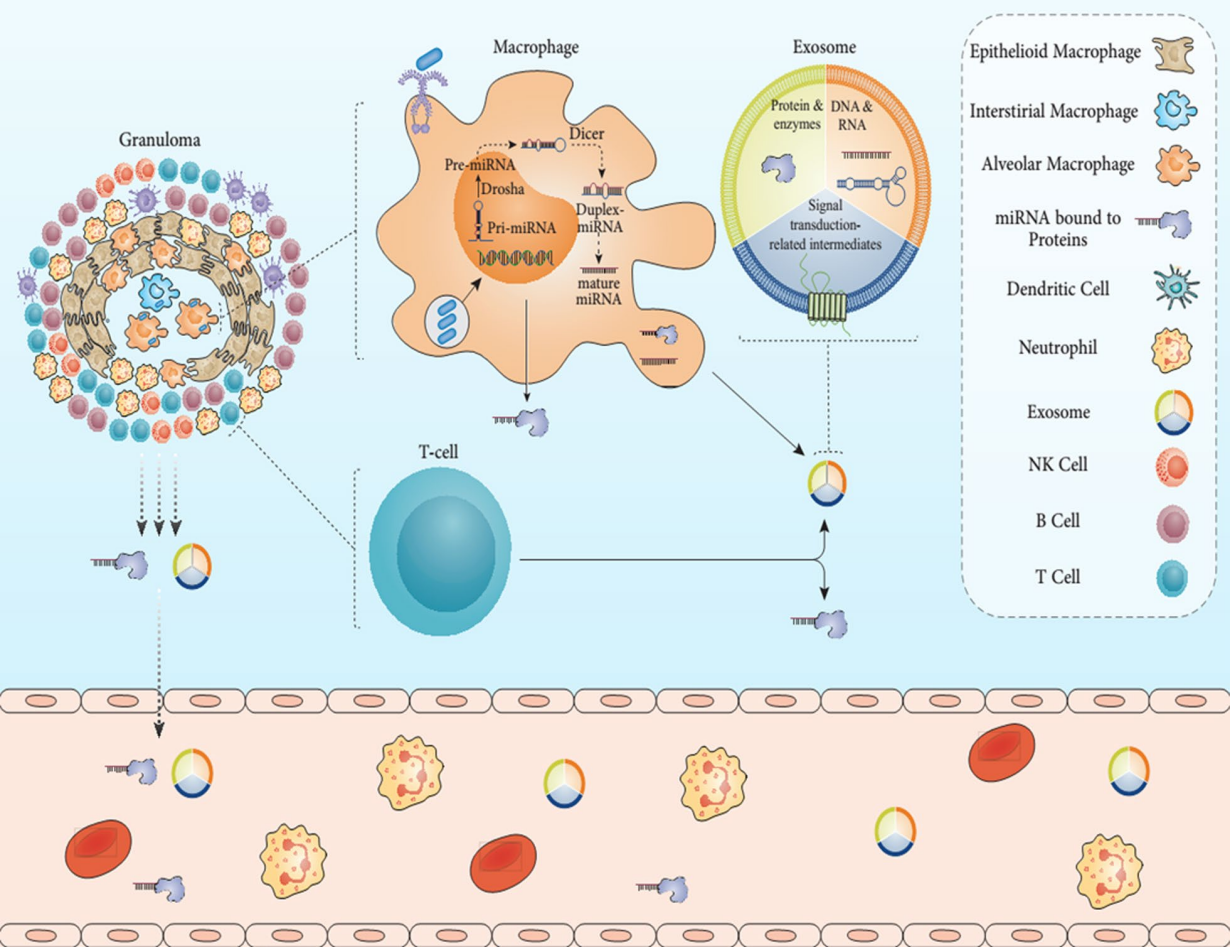
Exosomal miRNA in tuberculosis. Mtb PAMPs are identified by TLRs and other pattern recognition receptors, which result in the enhanced expression levels of primary-miRNAs in macrophages. In the nucleus and cytoplasm, these transcripts are cleaved by Drosha and Dicer; sequentially, as a result, the mature miRNAs (18–22 nucleotide) formed and acted to fine-tune intracellular immune processes. The varying miRNA subsets may have a vital role in regulating the particular pathways and components of the immune reactions. Simultaneously, adjacent T lymphocytes implicated in granuloma development/maintenance enhanced T cell subset particular miRNAs as a mechanism of tempering the type of adaptive immune response. Afterward, by the way, not yet entirely comprehended, these extracellular miRNAs proceed from local infection places to the circulatory system. This manner can consequently give rise to infection-specific miRNA expression signatures in circulating that can quickly be evaluated from serum, plasma, sputum, and other biological fluid (Correia et al. 2017). Mtb: *Mycobacterium tuberculosis*; PAMPs: pathogen-associated molecular patterns; TLRs: toll-like receptors

Researches on exosomal RNAs in various fields such as tumor biology, immunology, and neurobiology have shown that they may be employed as molecular targets toward many disorders (Fig. 3) (Schorey and Bhatnagar 2008; Skog et al. 2008). Although exosomes' function in the transportation of genetic matter, primarily miRNA, in TB amidst clinical humankind has not been established, comprehensive proteomic investigation described the protein content of MQs-derived exosomes infected with Mtb or treated Mtb CFP and mice infected with Mtb (Giri et al. 2010; Kruh-Garcia et al. 2014). These exosomes found in host proteins and mycobacterial proteins were shown to support innate and adaptive immune reactions in vitro and in vivo (Bhatnagar et al. 2007; Giri and Schorey 2008; Giri et al. 2010; Cheng and Schorey 2013). Exosomes produced from *Mycobacterium avium* infected MQs comprise pathogenic microbial glycopeptidolipids that induce pro-inflammatory reactions (Bhatnagar and Schorey 2007). Pioneering experiments have established RNA's quality in exosomes, and genetic material might be distributed among cells (Gusachenko et al. 2013). These RNAs are remarkably enduring to protect against enzyme degeneration in body fluids (Alipoor et al. 2016b,^2017^; Furci et al. 2013), recommending the practical and distinguishing capacity of these exosomal RNAs in TB (Valadi et al. 2007; Cheng et al. 2014; Eissa 2013). In precise, exosome-derived miRNAs have been explained to manage gene expression and cell activity in vitro and in vivo (Ismail et al. 2013; Chen et al. 2014). Singh et al. (2015a) represented the signature of host-related miRNAs, and mRNA copies and mycobacterial RNAs in exosomes originated from MQs infected by Mtb (Table 2). However, there is still a shortage of RNA-seq dependent research on human clinical samples. The current study determined the patterns of exosomal mRNA signs in normal subjects, LTBIs, and active TB cases (Lv et al. 2017). In a study, Lyu et al. (2018) have used the RNA-seq method for a limited RNA library to investigate different exosomal miRNA patterns in sera of healthy subjects, LTBI, and TB (Table 3). They exhibited different expression patterns of exosomal miRNA, suggesting the optional loading of miRNA toward exosomes during various physiological conditions (Lyu et al. 2018). Lyu et al. (2018) also showed six different miRNA expressions and three elevated continuous miRNAs such as hsa-miR-3184-5p, hsa-miR-140-3p hsa-miR-423-3p were monitored as possible mediators in progression TB.

**Table 2:** Exosomal miRNAs in tuberculosis

Exosomal miRNA	Source	Conclusion	Method for exosomes	Refs
miR-484, miR-425, and miR-96	Serum	The findings of this research indicate that exosomal miRNAs have a potential capacity for the diagnosis of active TB	Total exosome isolation (TEI) reagent (Invitrogen, Thermo Fisher Scientific Corporation, USA)	Mortaz et al. (2019)
miR-20a, miR-20b, miR-26a, miR-106a, miR-191, miR-486	Plasma	This study's results have indicated that the combination of exosomal miRNAs and EHRs might enhance the medical diagnosis of TBM and PTB	ExoQuick Kit (System Biosciences, USA)	Hu et al. (2019)
hsa-let-7e-5p, hsa-let-7d-5p, hsa-miR-450a-5p, and hsa-miR-140-5p, hsa-miR-1246, hsa-miR-2110, hsa-miR-370-3P, hsa-miR-28-3p, and hsa-miR-193b-5p	Serum	This research's outcomes recommended that exosomal miRNA use could promote novel molecular targets to distinguish LTBI and active TB	Differential centrifugation, filteration, and ultrafiltration	Lyu et al. (2019)
hsa-miR-140-3p, hsa-miR-3184-5p and hsa-miR-423-3p	Serum	The study’s findings imply the preferential packing of RNA loads for exosomes at various steps of Mtb infection and promote further analysis and production of TB pathogenesis	Differential centrifugation, filteration, and ultrafiltration	Lyu et al. (2018)
miR-1224, -1293, -425, -4467, -4732, -484, -5094, -6848–6849, -4488 and -96	PBMCs	This study's findings offer support for the release of unique exosomal miRNAs from BCG-infected MDMs, which highlighted the interaction between host and pathogen following infection	Total exosome isolation (TEI) reagent (Invitrogen by the Thermo Fisher Scientific Corporation, Waltham, MA, USA)	Alipoor et al. (2017)
Over 100 transcripts were found, such as 99b-5p, Mmu 30c, Mmu 30a, Mmu 191, Mmu 378, Mmu 210, Mmu 423-5p and Mmu 486-5p	Cell culture, Mtb infected RAW264.7 macrophages	This study has shown that there is discriminating packing of RNA material in exosomes after Mtb infection	Centrifugetion, filtration and linear sucrose gradient	Singh et al. (2015a)
miR-205-5p, miR-483-5p, miR-375, miR-200c-3p, miR-429, miR-200b-3p, miR-200a-3p, miR-203a-3p, and miR-141-3p	Pleural effusion	This study's outcomes have revealed that distinct miRNA profiles could be promising as biomarkers for differential determination of PEs with further confirmation dependent on larger cohorts	Differential centrifugation, filteration, and ultrafiltration	Wang et al. (2017)

TB, Tuberculosis; HERs, electronic health records; PTB, pulmonary tuberculosis; TBM, tuberculous meningitis; LTBI, latent TB infection; TEM, Transmission electron microscopes; Mtb, *Mycobacterium tuberculosis*; PBMCs, Peripheral Blood Mononuclear Cells

**Table 3:** Exosomal miRNA during active versus latent stage of tuberculosis and their implication in the different stages

Exosomal miRNA	Source	Changing trend	Active tuberculosis	Latent tuberculosis	References
miR-484, miR-425, miR-96	Serum	Up	The expression of miR-484, miR-425, and miR-96 was significantly increased in serum of TB patients, which correlated with the TB infection level. These results demonstrate that exosomal miRNAs have diagnostic potential in active tuberculosis	–	Alipoor et al. (2019a)
hsa-let-7e-5p, hsa-let-7d-5p, hsa-miR-450a-5p, and hsa-miR-140-5p	Serum	Up	–	In a study, in LTBI, hsa-let-7e-5p, hsa-let-7d-5p, hsa-miR-450a-5p, and hsa-miR-140-5p were found. Some members of the hsa-let-7 family were reported to play roles in the immune response to Mtb infection	Fu et al. (2011), Lyu et al. (2019)
hsa-miR-1246, hsa-miR-2110, hsa-miR-370-3P, hsa-miR-28-3p, and hsa-miR-193b-5p	Serum	Up	In TB samples hsa-miR-1246, hsa-miR-2110, hsa-miR-370-3P, hsa-miR-28-3p, and hsa-miR-193b-5p 5p were found. These specifically expressed miRNAs and differentially expressed miRNAs in different panels and patterns provide potential biomarkers for the detection/diagnosis of latent and active TB using exosomal miRNAs	–	Lyu et al. (2019)
miR‐148a‐3p, miR‐150‐5p, and miR‐451a	Pleural effusion	Up	A study found that miR-148a-3p, miR-451a, and miR-150- could distinguish TB from benign lesions. Hence these miRNAs barely searched in TB, which prompted new biomarkers in the diagnosis of TB	–	Wang et al. (2017)
miR-20a, miR-20b, miR-26a, miR-106a, miR-191, miR-486	Plasma	Up	In a study identified 6 exosomal miRNAs (miR-20a, miR-20b, miR-26a, miR-106a, miR-191, miR-486) in TB patients, 3 out of which (miR-20b, miR-191 and miR-486) showed a significant discriminatory value for pulmonary TB (PTB), TB meningitis (TBM)	–	Hu et al. (2019)

Additionally, they co-evaluated the distinctively expressed miRNA pattern with distinctively expressed mRNA patterns. They have described exploring the activity of exosomal RNA in the pathogenesis possess of TB (Lyu et al. 2018). Their conclusions presented relevant information on exosomes' potential role throughout the Mtb contagious process and boosted acknowledging exosomal miRNAs as possible biomarkers in diagnosing TB.

Lyu et al. (2018) defined several different down-regulated and up-regulated miRNA expression profiles in the current study. They analyzed the top ten miRNAs in each panel of the three classes. Earlier experiments have explained miRNAs distributed miRNAs expressed distinctively in plasma and serum as possible biomarkers for diagnosing TB. Despite this, most miRNAs in serum or saliva in humans are incorporated into the exosomes that can be shielded from enzymatic degeneration. Lyu et al. (2018) evaluated their analytical findings by comparing them to others. Qi et al. (2012) (Gallo et al. 2012; Lyu et al. 2018) declared that some miRNA such as miR-576-3p might distinguish TB patients from healthy subjects with reasonable sensitivity and specificity. Frequently, Lyu et al. (2018) have shown that miR-576-3p was clearly increased in TB cases relevant to healthy controls. Although, in their analysis, Lyu et al. (2018) demonstrated a decrease in expression of miR-483-5p and enhanced expression of miR-486-5p in cases with TB, which was reversed in other published research (Zhang et al. 2013,2014). Additionally, couples with distinctively expressed miRNA patterns have been mentioned as a potential prognostic marker for TB. Some of the miRNAs in (Lyu et al. 2018) investigations in the corresponding miRNA families such as miR-29a-3p, miR-93-5p, miR-378d, miR-378i, miR-378i, miR-22-3p, miR-155-5p, and miR-196b, have also been identified in the same administrative profile in TB subjects.

In comparison, miR-let-7e-5p was enormously increased in LTBI relative to healthy controls but was substantially decreased in TB contrasted to LTBI (Lyu et al. 2018). It has recently been identified the MiR-let-7 implicated in apoptosis of cancer cells and its innovative roles in regulating immune reactions against Mtb (Fu et al. 2011; Shimizu et al. 2010). The current investigation shows that exosome-embedded miRNAs in the breathed inhalation have a promise biomarker development in cases with pulmonary infections such as TB. These distinct miRNAs highlight characteristic findings of LTBI biomarkers and TB determination (Sinha et al. 2013).

In a report, Alipoor et al. (2017) discovered that human monocyte-derived MQ (MDM) infection with *M. bovis BCG* mediated the release of a complex group of exosomal miRNAs implicated in modulating main metabolic and energy formation pathways, as well as in the modulation of immunological and cell signaling events. Hence in another study, Alipoor et al. (2019c) speculated the percent exosomal miRNAs secreted from infected cells with Mtb could have the sense to be prosperous diagnostic and therapeutic biomarkers. In small pilot research, the miRNA expression such as miR-425, miR-484, and miR-96-3P that attenuate these fundamental mechanisms in serum exosomes in subjects with TB was assessed to estimate its possibility as a diagnostic and dynamic standing biomarker for TB (Alipoor et al. 2019c). In this pilot study, Alipoor et al. (2019c) demonstrated some miRNA levels in exosomes enhanced, including miR-425, miR-484, and miR-96 in serum exosomes in TB cases and subgroup examination, which exhibited that miRNA levels were correlated with the bacterial burden. The research has revealed that individual serum exosomal miRNAs have an acceptable predictive value for TB. However, this predictive value advances entirely based on the rise in the quality of smear positivity utilizing miR-425 and miR-484, as well as the mixture of miRNA expression levels (Alipoor et al. 2019c). In vitro, BCG infection of human MQs promotes exosomal disclosure of 11 miRNAs implicated in managing many principal host pathways, including metabolic processes, cell signal transduction, and infectious disease pathways resulting in energy formation intracellular bacterial persistence (Alipoor et al. 2017). Since the resemblance between the BCG-and Mtb-induced miRNA patterns has been documented recently, Alipoor et al. (2019c) designated a subcategory of these expression profiles' miRNAs as miR-484, miR-96, and miR-425, to determine if these serum exosomal miRNAs might serve as a possible biomarker in TB patients. Preliminary determination of circulating miRNAs in serum in response to Mtb infection shows that the 92 circulating miRNAs in serum were substantially different in TB cases relative to healthy subjects (Fu et al. 2011; Yi et al. 2012). Yi et al. (2012), repeatedly employing an array-based system, identified a bunch of 95 miRNAs expressed separately in subjects with TB sputum relevant to control subjects.

In a report, Lyu et al. (2019) examined expression patterns of small RNA (particularly miRNA) in exosomes (in serum sample) originated from LTBI and TB cases and compared them with controls. MiRNA patterns of exosomes from LTBI, TB, and healthy control groups were established, recommending the preferential loading of miRNA loads into exosomes at distinct phases of Mtb infection (Lyu et al. 2019). In particular, Lyu et al. (2019) described several explicitly expressed miRNAs. The expression profiles of miRNAs came from different panels, and trends in the three classes presented possible biomarkers to determine LTBI and active TB employing small RNA, especially exosomal miRNAs. From the repeated genomic sequences, many small RNAs derived, including long interspersed nuclear elements (LINES), long terminal repetition (LTR), and short interspersed nuclear elements (SINES), have also been seen that may play a function in the induction of host immune reactions concurrently with Mtb infected advancement (Lyu et al. 2019). Their results provide strong evidence and a better comprehension of miRNAs and constant region-originated small exosome RNAs throughout the Mtb disease process and promote the progress of prospective molecular mechanisms to distinguished LTBI active TB (Lyu et al. 2019). In the current study, Lyu et al. (2019) demonstrated differential expression patterns of miRNA in serum exosomes obtained from the latent, active TB, and healthy subjects groups. Primarily, they recognized some explicitly expressed miRNAs in all groups of distinctively expressed miRNAs, and they offer a possible role as biomarkers for the identification of LTBI and active TB. Besides, in the TB sample, some exosomal miRNAs, including hsa-let-7d-5p, hsa-let-7e-5p, hsa-miR-140-5p, and hsa-miR-450a-5p in latent patients and some miRNAs in active patients such as hsa-miR-2110, hsa-miR-1246, hsa-miR-28-3p, hsa-miR-370-3P, and hsa-miR-193b-5p were verified to be accurately expressed (Lyu et al. 2019). Besides, three explicitly articulated miRNAs referring to the human miRNA lethal family 7 (hsa-let-7) were distinguished in LTBI groups: hsa-let-7d-5p, hsa-let-7d-3p, as well, as hsa-let-7e-5p (Lyu et al. 2019). Parts of the hsa-let-7 family have been documented to perform an essential role in the immunity to Mtb infection (Fu et al. 2011; Sharbati et al. 2011). Several explicitly expressed miRNAs like hsa-miR-144-3p, hsa-miR-142-3p, as well as hsa-miR-23a-5p have been documented to be intimately connected to the immune reaction to Mtb disease:

- Hsa-miR-142-3p can attenuate phagocytosis by interfering with Neural Wiskott-Aldrich Protein Syndrome (N-Wasp) in host cells.
- MiR-144-3p can hinder autophagy activity, and via targeting ATG4a in RAW264.7 MQ cells promote BCG infection.
- MiR-23a-5p might manipulate autophagy and Mtb persistence by affecting TLR2 in TLR2/MYD88/Nuclear factor-kappa B (NF-κB) pathway during the Mtb infection phase (Bettencourt et al. 2013; Guo et al. 2017; Gu et al. 2017).

Exosomes released from Mtb-infected MQs or mycobacterial culture-treated filter proteins and Mtb-isolated exosomes-infected mice have been distinguished for their protein content. They hold to help both the immune system's arms for induction (innate and adaptive immune responses) in vitro and in vivo (Giri et al. 2010; Giri and Schorey 2008; Cheng and Schorey 2013). However, it is not known if the RNA found in these exosomes relates to this immune reaction. To resolve this dilemma, it is crucial to discriminate the RNA content inside the exosomes; thus, Singh et al. (2015b) report that screened exosomes are released from Mtb-infected MQs, and exosomes were found to include host miRNAs and messenger RNAs. Although a general decreased amount of host miRNAs was discovered in exosomes from infected cells, a group of miRNAs and mRNA transcripts specific to these exosomes were also identified (Singh et al. 2015b). The findings indicate that there is preferential packing of RNA material in exosomes following Mtb infection. The exosomal RNA might be passed to and converted into the receiver cells, which could induce a biological reaction in these cells. Singh et al. (2015b) have found mycobacterial transcripts in exosomes discharged from MQs infected with Mtb and EVs extracted from the TB patient's serum. To our knowledge, Singh et al. (2015b) is the initial investigation to demonstrate the existence of pathogen-obtained RNA in exosomes generated throughout bacterial infection. MiRNAs may be implicated in controlling gene expression for pathways essential to the immune reactions to infection. Variations in circulating miRNAs in subjects with TB serum relevant to controls have been recognized, and these variations may render signatures to discriminate active from latent TB (Wang et al. 2011). While the investigators have not directly looked at exosomes, it is understood that the bulk of miRNAs in human serum or saliva are encompassed in exosomes that improve the viability of the RNA (McDonald et al. 2014). Lately, exosome-embedded miRNAs in exhaled ventilation have been recommended as possible biomarkers for cases with respiratory infections such as TB (Gallo et al. 2012). In initial studies, Singh et al. (2015b) based on miRNAs embedded in exosomes generated from Mtb-infected MQs and how this is related to non-infected cell exosomes. To investigate this problem, they followed a sequencing method for the detection of miRNAs in exosomes.

While they detected a group of miRNAs that were unique to exosomes from infected cells, most miRNAs isolated were found in both infected and uninfected MQs, indicating general preservation in the transportation and integration of miRNAs into exosomes (Singh et al. 2015b). In sum, Singh et al. (2015b) reported 57 miRNAs in exosomes generated from infected MQs, such as Mmu 223 and 486-5p, which relate to a cluster of expression profiles miRNAs in patients with TB serum. Earlier studies have also shown that miRNA 99b is firmly up-regulated in Mtb infected DCs and aims TNF-α and TNF receptor superfamily (TNFRSF)-4 receptor gene transcripts and that less abundance of this miRNA results in a substantial loss of microbial viability in DCs (Singh et al. 2013). Mycobacterial discharged protein ESAT6 is also a recognized miR-155 effector whose up-regulation is accompanied by Mtb infection attenuates a group's expression of proteins that gain from infection (Kumar et al. 2012). These findings indicate that cellular miRNAs are more numerous after infection with Mtb can provide a mechanism for immune escape by the pathogen. Interestingly, in their quantitative reverse transcription-polymerase chain reaction (RT-PCR) tests, Singh et al. (2015b) observed that the number of these and other miRNAs declined substantially in exosomes that released Mb-infected relative to non-infected cells. MiRNAs described were engaged in diverse pathways, including Calcium signaling, mitogen-activated protein kinase (MAPK) signaling, NK-mediated cytotoxicity, and Janus kinase (JAK) signaling transducer and transcription activator (STAT) signaling, all of which were implicated in the immune response to infection (Singh et al. 2015b).

In a report, Wang et al. (2017) in lung adenocarcinoma investigate expression patterns of exosomal miRNAs distinctively in pleural effusion (PE), TB, and other benign lesions (NPE) employing deep sequencing and qRT-PCR. Accurate analysis of the results revealed that quite a few miRNAs vary between three groups of PEs; in particular, some miRNAs were notably widely raised in one group (Wang et al. 2017). In the Wang et al. (2017) report, miRNAs such as miR-200b-3p, miR-200c-3p, miR-200a-3p, miR-429 were the most prevalent profiles relative to TB, and miR-205-5p, miR-141-3p, miR-375, miR-483-5p, and miR-203a-3p were the most prevalent adenocarcinoma profiles relative to NPE promised to be used to differentiate adenocarcinoma from other disease states with further verification. However, TB and NPE had the almost identical and weak expression of miRNAs relevant to adenocarcinoma. Only three slightly individual miRNAs, such as miR-148a-3p, miR-451a, and miR-150-5p, promised to separate TB from NPE (Wang et al. 2017). Five of the nine miRNAs raised a substantial difference in adenocarcinoma relevant to others. Former investigators have urged that miR-200b-3p, miR-200c-3p, miR-200a-3p, miR-141-3p, and miR-429 belong to the miR-200 family, and all five parts are managed in cells sustaining epithelial-mesenchymal transformation (Wang et al. 2017). In summary, exosomal miRNA expression profiles vary among lung adenocarcinoma, TB, and benign specimens. Findings of Wang et al. (2017) reported a total of nine miRNAs are selectively categorized as adenocarcinoma-derived exosomes such as miR-375, miR-205-5p, miR-483-5p, miR-429, miR-200c-3p, miR-200a-3p, miR-200b-3p, miR-141-3p and miR-203a-3p, as well as miRNAs such as miR-451a, miR-148a-3p, and miR-150-5p have differential expression between TB and NPE. Also, miR-375, miR-483-5p, and miR-429 were confirmed to be consistent with lung adenocarcinoma (Wang et al. 2017). These miRNAs can keep a guarantee as biomarkers to determine PEs with verifying in more extensive cohort investigations.

In a pilot experiment, Alipoor et al. (2017) in human MQs following co-infection with *M. bovis* BCG identified the exosomal miRNA. They presumed that MQs infected with BCG could release a particular collection of exosomal miRNAs that could participate in TB pathogenesis. Alipoor et al. (2017) were found in 11 exosomal miRNAs such as miR-1293, miR-1224, miR-425, miR-4732, miR-4467, miR-484, miR-6848, miR-5094, miR-96, miR-6849, and miR-4488 expressed differently in infected cells. These miRNAs are implicated in various principal pathways, such as central carbon metabolism, sugar metabolism, fatty acids, amino acid metabolism, cell signal transduction, and bacterial aggression mechanisms (Alipoor et al. 2017). This indicates that the host metabolic pathways involved in protective immunity are mitigated to facilitate MQ's microbial persistence. Exosomal miRNAs can impair fatty acid synthesis and correlated metabolites following BCG therapy (Alipoor et al. 2017). Consequently, miR-1224 is one of the disturbed exosomal miRNAs discovered in the Alipoor et al. investigation (2017).

Consequently, miR-1224 is one of the disturbed exosomal miRNAs discovered in the Alipoor et al. investigation; this miRNA is engaged in controlling lipid metabolism. The expression of lipid-mediated genes under the control of MiR-1224 via regulating transcription factors, including protein specificity 1 (SP1) (Vickers et al. 2013). In this way, in cases with TB, investigations have been confirmed an excess of lipids in caseous pulmonary granulomas (Kim et al. 2010). The transcriptome investigation findings show a substantially elevated expression of genes participating in the host lipids biosynthesis, sequestration, and catabolism (Kim et al. 2010). Moreover, a rise in the amount of TNF-α following exposure to Mtb cell wall elements promotes the triggering of lipid metabolism-related genes in infected cells (Kim et al. 2010). The TNF-α gene expression hinders by MiR-1224, which designates an intimacy among these pathways (Niu et al. 2011).

All in all, the modification of the lipid metabolism of the host plays a critical function in the preservation of intracellular Mtb infection (Kim et al. 2010; Daniel et al. 2011; Peyron et al. 2008). Mtb employs the host's fatty acids as a carbon supply and reduces propionyl-CoA's impact, an intensely poisonous intermediate, on its own endurance (Alipoor et al. 2017). Another category of distinctively expression patterns of miRNAs in exosomes-originates from infected MQs is correlated with the cell membrane and contact pathways like an adhesive junction, void junction, heparan sulfate/keratin sulfate metabolism, and glycosaminoglycan biosynthesis (Alipoor et al. 2017). For instance, miR-1293 targets metalloproteinase tissue inhibitors (TIMPs) (Li et al. 2013). The matrix metalloproteinase (MMP) inhibitor called TIMP-1 is active in the aggression and dissemination of bacteria by epithelial cells (Friedland et al. 2002). Following Mtb infection, the expression of MMP was elevated and disrupted the MMP/TIMP equilibrium in monocytes (Elkington et al. 2005). In MQs infected with BCG, the expression of miR-1293 was raised to denote mycobacterium Ags' capacity to modify the host cell membrane's composition and ultimately affect the MQ's longevity cells (Alipoor et al. 2017). MiR-484 and miR-425 have been identified ideally in BCG-infected MQ cells. Intermediate metabolic from metabolic pathways controlled by MiR-484 via engaging mitochondrial fission protein 1 (Fis1) and modified miR-425 levels are consistent with insulin resistance (Wang et al. 2012). The role of these miRNAs in MQs infected persists uncertain at the stage mentioned above, although it is apparent that miR-425 controls multiple metabolic processes and is correlated with metabolic diseases (Barwari et al. 2016). MiR-1224, miR-1293, miR-4732, miR-4467, miR-5094, miR-6848, and miR-6849 are human mirtrons formed by the splicing of mRNA coding introns than by the forming of Drosha hairpin loops (Butkytė et al. 2016). These mirtrons' expression was substantially higher in exosomes originated from infected MQs (Alipoor et al. 2017; Butkytė et al. 2016). This indicates that mycobacteria can somewhat partially recognize the expression patterns of miRNA development inside the host cell and process the over-production of these types of miRNA called mirtrons to hire host biological processes that favor Mtb disease.

Monocytes were harvested from human blood and then conveyed to the MDM phenotype employing conventional protocols (Mortaz et al. 2016). MDMs were infected with *M. bovis* BCG or willed as non-infected control. 72 h after infection of cell culture median, RNA isolating and exosomes were harvested. In this analysis, the libraries' small RNA was developed, and RNA sequences were conducted. Raw reads filtered to reduce adapter and primer sequences. FASTQ sequences were run towards miRNA sequences of humans in miR Base, employing BLAST tools applying the Linux operating method (Mortaz et al. 2016). They observed that BCG MDM infection contributed to the secretion of various exosomal miRNAs, including the members of the Let-7 family, miR-155, miR-145, miR-146a, and miR-21, which were all supposed to target critical immune-correlated genes and pathways (Mortaz et al. 2016). This research offers proof of the secretion of unique miRNAs from MDMs infected with BCG. However, these findings and the involvement of this miRNAs profile evaluated in patients' blood should be checked to assess their selectivity and distinction as determinations of TB cases.

In this same Alipoor et al. (2019b), relying on this finding, human MDM infections with BCG stimulated the release of a specific collection of exosomal miRNAs, include mir‐1224, mir‐484, mir‐1293, mir‐423, and mir‐96, which are implicated in modifying primary host metabolic and energy development pathways and also in the modulation of immunological and cell signaling processes. In the current research, the possible regulatory impact of the group of dysregulated exosomal miRNAs generated by human MDMs on Mycobacterium infection was examined (Alipoor et al. 2019b). Computational biology has shown that these miRNAs control critical metabolic and resource pathways such as central carbon metabolism, sugar metabolism, fatty acids, bacterial aggression, immunological process, and cell signaling pathways (Alipoor et al. 2019b). This conclusion designates that Mycobacterium can employ host miRNAs to attenuate host metabolic reprogramming or re-patterning for intracellular viability. These dysregulated miRNAs could be embedded within exosomes and discharged into body fluid to modulate local and distal cells' activity. Results of Alipoor et al. (2019b) designated a total of 1022 connection pairs across 96 nodes. Cluster one was the most adjusted with the maximum score. Two or five other clusters of nodes improved with metabolism and infection-related pathways. This intimates that these miRNAs and their gene targets have comprehensive associations to control the host resistance to Mycobacterium infection. Relevant proteins targeting these clusters contain mitochondrial fission protein one controlled by miR‐484 and various lipid metabolizing proteins targeted by miR‐1224 (Vickers et al. 2013; Wang et al. 2012). Besides, miR‐425 has also been active in regulating multiple metabolic processes and is correlated with many metabolic diseases (Barwari et al. 2016). Also, both miR‐425 and miR‐96 intercede insulin resistance with miR‐96, which controls the expression of diverse genes that release fine-tune insulin (Chakraborty et al. 2013; Yang et al. 2016). For instance, miR‐96 represses insulin discharge by increasing the amount of granuphilin, an insulin exocytosis inhibitor (Lovis et al. 2008). MiR‐96 is highly caused by fatty acids and decreases insulin receptor expression (INSR) and INSR substrate 1 (Yang et al. 2016). On the other side, Mtb-infected MQs exhibit reduced sugar supply and significant increases in intracellular glucose, glycogen, nicotinamide adenine dinucleotide (NAD)/nicotinamide adenine dinucleotide phosphate (NADP), and lactate amounts (Mehrotra et al. 2014; Amila et al. 2015). Mtb infection often induces enhanced aerobic glycolysis due to pentose-phosphate shunt and increased circulating absorption (Mehrotra et al. 2014; Ndzi et al. 2019).

As exosomal miRNAs appeared as potential biomarkers, Hu et al. (2019) explored the potential for exosomal miRNAs and electronic health records (EHRs) to be used to diagnose TB. Three hundred seventy cases comprising TB meningitis (TBM), pulmonary TB (PTB), non-TB illness management, and stable condition controls were registered in the sample. Exosomal miRNAs were recorded in the exploration cohort using microarrays. miRNAs applicants were chosen in the selection cohort using qRT-PCR, and EHRs and patient follow-up details were also obtained (Hu et al. 2019). Exosomal miRNAs such as miR-20a, miR-26a, miR-20b, miR-191, miR-106a, and miR-486 were differentially expressed in TB cases (Hu et al. 2019). The conclusions demonstrate that the effectiveness of exosomal miRNAs and EHRs might enhance TBM and PTB's medical assessment. To sum up, the findings of Hu et al. (2019) recommend that exosomal miRNAs such as miR-191, miR-20b, and miR-486 are prospective miRNA as diagnostic biomarkers for TB. Combining data from miRNA and EHR data via a machine learning algorithm could be a reasonable strategy to enhance TBM and PTB's differential analysis and demand verification in larger groups.

## Exosomal RNAs as a diagnostic and therapeutic biomarker for tuberculosis

Exosomes have recently arisen as an essential domain in human disorders and infectious pathogens linked to their material, comparative facility of separation, and immunoregulation features (Hadifar et al. 2019). Determination, vaccine, and care dependent on potential biomarkers role for exosomal have also gained substantial interest in different diseases. For example, various exosome-based vaccine applicants have been tested at many levels of clinical trials, and exosome stability has been established (Chaput and Théry 2011; Dai et al. 2008; Escudier et al. 2005). It seems that the perception of exosomal proteome might be beneficial for this purpose. Several molecules include RNAs, have been identified in the exosomal element contains miRNA, mRNA, ribosome RNA (rRNA), and long non-coding RNA (Hadifar et al. 2019). Exosomal RNAs (particularly miRNAs) seem to be the perfect platform for producing diagnostic biomarkers due to the durability of exosome-mediated resistance towards RNA deterioration (Hunter et al. 2008).

Current data recommending mycobacterial proteins' composition in infected MQs drove exosomes were obtained mainly from western blots (Kruh-Garcia et al. 2015). A systematic proteomic questionnaire was conducted for MQs -derived exosomes dealing with live and dead Mtb (g-irradiated Mtb) to support and expand on these results (Giri et al. 2010; Kruh-Garcia et al. 2015). Preliminary proteomic exploration investigations have shown that these exosomes' proteome is fundamentally made up of host proteins with about 1% of the spectroscopy data obtained to distinguish mycobacterial peptides (Giri et al. 2010; Kruh-Garcia et al. 2015). More than 40 Mtb proteins identified in exosomes of MQs infected with live Mtb compared with g-irradiate Mtb exhibited exosomes completed in a single match of the Mtb peptide according to the results of liquid chromatography–tandem mass spectrometry (LC–MS/MS) (Giri et al. 2010). Furthermore, the distinguished proteins were mainly formed of recognized discharged and fully antigenic proteins, such as the Ag 85 complex (Rv3804c, Rv1886c, Rv0129c), Mpt32 (Rv1860), and HspX (Rv2031c) (Målen et al. 2007; Rosenkrands et al. 2000). Compatible with these findings, the enumeration of Mtb proteins in the host’s exosomes throughout the disease can rely on live microbes being phagocytized. It could involve active intracellular excretion, presumably through SecA, or additional excretion ways, like Type VII excretion (Kruh-Garcia et al. 2015).

Singh and co-workers (2015a) evaluated RNAs in exosomes derived from bacteria. In their analysis, Mtb's RNA profiling in infected MQ originating from exosomes presented unique mRNA fingerprints and preferential exosomal labeling of host-mRNAs -miRNAs and TB RNAs in Mtb infection (Singh et al. 2015a). Recent findings on exosomal RNAs indicated that they could have potential diagnostic tools for Mtb disease. According to several reports, exosomal miRNAs can be identified in the infection process (Skog et al. 2008; Rabinowits et al. 2009). Wang and colleagues indicate that throughout pleural effusion, benign wounds, and pleural effusion of TB, the distinctive pattern of expression of exosomal miRNAs (like miR‐148a‐3p, miR‐150‐5p, and miR‐451a) can act as potential biomarkers for TB pathology (Wang et al. 2017). The mRNA characterization of exosomes obtained from serum samples of healthy subjects and active and LTBI cases indicated preferential exosomal RNA packing during various physiological conditions about an exclusive enhancement of specific RNAs in latent TB infection (Lv et al. 2017). Lv and co-workers (2017) investigate the exosomal RNA biomarker's role in the differential diagnosis of LTBI and active TB by analyzing various genes' expression patterns. Besides, they (Lyu et al. 2018) investigate the sera of patients with LTBI and active TB to determine patterns of expression of exosomal miRNA. The results indicate that miRNAs expressions in both were significantly different (Lyu et al. 2018). The Lv et al. result suggests that the miRNAs in exosomes were implicated in TB's development and pathogenesis, so they proposed that this miRNA can serve as biomarkers for TB diagnosis.

The inherent biomarkers role of exosomal miRNAs for TB diagnosis has been evaluated in additional experiments (Alipoor et al. 2017). Evidence has been provided for various expression levels of exosomal miRNAs (Table 1) in MQs infected with *M. bovis* BCG (Hadifar et al. 2019). In the pathogenesis of TB, these exosomal miRNAs can play a significant role and possibly uses as TB markers for diagnosis. The evidence obtained cast doubt on exosomes' work and the possible involvement of exosomal RNAs as innovative biomarkers for TB in the advent of new diagnostic methods in the context of TB. Nevertheless, the use of exosomal RNAs as an innovative origin of biomarkers has several criteria, include reliable and worthwhile insulation procedures for low-level specimens and systematic RNA study programs. To date, the BCG remains the only licensed vaccine against TB, with more than 90% coverage in countries with high TB incidence (Mangtani et al. 2014; Zwerling et al. 2011; Fine 1995). Besides the protective role of BCG against TB infection, the BCG can interfere with diagnosing active TB and LTBI using the purified protein derivative (PPD) in tuberculin skin test (TST) Mantoux. The intradermal injection of PPD produces a type IV hypersensitivity reaction and is applied to diagnose both active and LTBI (Pahal and Sharma 2020). After BCG vaccination, the diagnostic value of PPD’s reduced because the PPD has a low specificity. This is because PPD is composed of many proteins from Mtb (more than a hundred proteins). Various of them are being in BCG, too, cause antigenic cross-reactivity and inducing a high degree of false positivity (Kasempimolporn et al. 2019). A reduction in the antigen cross-reactivity is needed for test precision, despite the limited investigation and discussions that have marked this dilemma (Whitlow et al. 2020).

Regarding the biomarkers or diagnostic role of exosomes in TB, researchers may be encountering some obstacles. Recent studies show that the BCG can induce the release of exosomes (Bhatnagar et al. 2007; Alipoor et al. 2017b). Hence, the BCG vaccination is possible provoking the release of exosomes, which can interfere with the potential biomarker function of exosomes in TB diagnosis.

## Concluding remarks

TB is known to be one of the most infectious and fatal diseases. It is a substantial public health issue worldwide since it is a drug-resistant infectious disease. Extraordinary instances are often latently infected with TB, and only a limited number of infected individuals will produce successful TB in their lifetime. Consequently, new diagnostic and therapeutic approaches are required to detect TB infection in the early stages of disease and TB infection management (mitigating TB transmission). The fundamental basis for this mess to eradicate TB is a paucity of knowledge of the molecular pathways causing TB pathophysiology and problems in its determination and therapy. EVs such as exosomes offer a hopeful investigation since they are discharged from several cells carrying useful biochemical evidence. As stated, the exosomal miRNA expression patterns in healthy subjects, LTBI, and TB were substantially different. Thus, couples with distinctively expressed miRNAs in both forms of the disease include TB or LTBI, could act as biomarkers to detect TB. Confirmation of these possible exosomal miRNAs in a clinically meaningful sample set can provide a basis for innovative TB biomarkers and advance our awareness of TB pathophysiology either development.

## Data Availability

Not applicable.
